# Phylogenomic analysis of the Chilean clade of *Liolaemus* lizards (Squamata: Liolaemidae) based on sequence capture data

**DOI:** 10.7717/peerj.3941

**Published:** 2017-10-26

**Authors:** Alejandra Panzera, Adam D. Leaché, Guillermo D’Elía, Pedro F. Victoriano

**Affiliations:** 1 Departamento de Zoología, Facultad de Ciencias Naturales y Oceanográficas, Universidad de Concepción, Concepción, Chile; 2 Programa de Doctorado en Sistemática y Biodiversidad, Universidad de Concepción, Concepción, Chile; 3 Department of Biology & Burke Museum of Natural History and Culture, University of Washington, Seattle, WA, United States of America; 4 Instituto de Ciencias Ambientales y Evolutivas, Universidad Austral de Chile, Valdivia, Chile

**Keywords:** Ultra-conserved elements, Liolaemids, Systematics, Chile

## Abstract

The genus *Liolaemus* is one of the most ecologically diverse and species-rich genera of lizards worldwide. It currently includes more than 250 recognized species, which have been subject to many ecological and evolutionary studies. Nevertheless, *Liolaemus* lizards have a complex taxonomic history, mainly due to the incongruence between morphological and genetic data, incomplete taxon sampling, incomplete lineage sorting and hybridization. In addition, as many species have restricted and remote distributions, this has hampered their examination and inclusion in molecular systematic studies. The aims of this study are to infer a robust phylogeny for a subsample of lizards representing the Chilean clade (subgenus *Liolaemus sensu stricto*), and to test the monophyly of several of the major species groups. We use a phylogenomic approach, targeting 541 ultra-conserved elements (UCEs) and 44 protein-coding genes for 16 taxa. We conduct a comparison of phylogenetic analyses using maximum-likelihood and several species tree inference methods. The UCEs provide stronger support for phylogenetic relationships compared to the protein-coding genes; however, the UCEs outnumber the protein-coding genes by 10-fold. On average, the protein-coding genes contain over twice the number of informative sites. Based on our phylogenomic analyses, all the groups sampled are polyphyletic. *Liolaemus tenuis tenuis* is difficult to place in the phylogeny, because only a few loci (nine) were recovered for this species. Topologies or support values did not change dramatically upon exclusion of *L. t. tenuis* from analyses, suggesting that missing data did not had a significant impact on phylogenetic inference in this data set. The phylogenomic analyses provide strong support for sister group relationships between *L. fuscus*, *L. monticola*, *L. nigroviridis* and *L. nitidus*, and *L. platei* and *L. velosoi*. Despite our limited taxon sampling, we have provided a reliable starting hypothesis for the relationships among many major groups of the Chilean clade of *Liolaemus* that will help future work aimed at resolving the *Liolaemus* phylogeny.

## Introduction

Lizards of the genus *Liolaemus* Wiegmann 1834 are widely distributed throughout temperate South America, occurring mainly in Argentina and Chile, but also in Bolivia, Brazil, Paraguay, Peru and Uruguay (Abdala & Quinteros, 2013). They cover a wide range of climatic regimes, from the Atacama Desert to the Valdivian rainforest, and from Mediterranean shrublands to Patagonian steppes (Donoso-Barros, 1966; Cei, 1986; Cei, 1993). *Liolaemus* has a long evolutionary history dating back to 18–22 million years ago (Schulte II et al., 2000; Fontanella et al., 2012). The genus is highly variable, exhibiting a wide range of reproductive modes, habitats, life histories, coloration patterns and body sizes (e.g., Koslowsky, 1898; Carothers et al., 1997; Schulte II et al., 2000; Ibargüengoytía & Cussac, 2002; Espinoza, Wiens & Tracy, 2004). Not surprisingly, *Liolaemus* has been subject to many ecological and evolutionary studies (e.g., Harmon et al., 2003; Camargo, Sinervo & Sites, 2010; Cianferoni et al., 2013; Sheldon, Leaché & Cruz, 2015). Nevertheless, phylogenetic relationships among species of *Liolaemus* have been difficult to resolve, due in part to the incomplete taxon sampling of most studies (but see Olave et al., 2014), the discordance between inferences based on molecular and morphological data (Schulte II et al., 2000; Lobo, 2001; Lobo, 2005; Vidal & Labra, 2008), and to the incongruences between nuclear and mitochondrial gene trees caused by, likely, incomplete lineage sorting and hybridization (e.g., Olave et al., 2011).

*Liolaemus* currently includes more than 250 species (Uetz, Freed & Hošek, 2016), although this is thought to be a rough underestimate (Morando, Avila & Sites, 2003). Since 2010, more than 40 new species have been described (Uetz, Freed & Hošek, 2016), and several phylogeographic studies have reported new candidate species (e.g., Cianferoni et al., 2013; Troncoso-Palacios et al., 2015; Olave et al., 2016), which suggests that the number of species will continue to increase. The genus is subdivided into two subgenera, *Liolaemus* (*sensu stricto*) and *Eulaemus* (Laurent, 1985), distributed mainly on the western and eastern sides of the Andes, respectively, although some species of the subgenus *Liolaemus* are found in the eastern slope of the Andes (e.g., in the *elongatus* group; Abdala & Quinteros, 2013). The monophyly of each subgenus has been strongly supported by both morphological and molecular data (Young-Downey, 1998; Schulte II et al., 2000; Espinoza, Wiens & Tracy, 2004; Morando et al., 2004). Each subgenus is also divided into several species groups, which are composed by presumably related species and are delimited based on morphological characters (such as lepidosis, coloration and body measurements) and life history traits (such as reproductive mode and type of habitat) (e.g., Pincheira-Donoso & Núñez, 2005; Lobo, Espinoza & Quinteros, 2010; Abdala & Quinteros, 2013). In Chile, the genus is represented by approximately 96 species (Ruiz de Gamboa, 2016). Studies attempting to resolve the phylogenetic relationships among Chilean species of *Liolaemus* are scarce compared to studies focused on Argentinean species (e.g., Camargo et al., 2012; Avila et al., 2013; Olave et al., 2011; Olave et al., 2014; Olave et al., 2015; Olave et al., 2016). In Chile, the low number of phylogenetic studies relative to the high diversity of *Liolaemus* has hampered the development of comprehensive biogeographical studies that in turn, may help to improve conservation strategies (Vidal & Díaz-Páez, 2012).

**Table 1:** Species groups recognized within the Chilean clade (*Liolaemus sensu stricto*) of *Liolaemus* indicating their species richness and number of species sampled. Adapted and updated from Abdala & Quinteros (2013). “*N*” indicates the number of species currently assigned to each species group, and “sampled” indicates the number of species sampled in this study. In the case of the *pictus* group four lineages were sampled that currently represent two described species (*Liolaemus pictus* and *Liolaemus tenuis*). See Table S1 for a detailed account of taxonomic rearrangements.

Section	Group	*N*	Sampled
*chiliensis*	*alticolor–bibronii*	26	2
	*bellii*	4	–
	*capillitas*	6	–
	*chillanensis*	3	–
	*elongatus*	17	–
	*gravenhorstii*	3	1
	*kriegi*	4	–
	*leopardinus*	5	–
	*pictus*	5	2
	*robertmertensi*	4	1
*nigromaculatus*	*monticola*	2	1
	*nigroviridis*	8	2
	*nigromaculatus*	11	5

Troncoso-Palacios et al. (2015) using three fragments of the mitochondrial genome inferred phylogenetic relationships for species of the subgenus *Liolaemus*; species were distributed into two main clades, named by the authors as the *chiliensis* and the *nigromaculatus* sections. However, species group were not recovered monophyletic. These results imply that current taxonomy does not reflect species evolutionary history and that a reappraisal is needed. Moreover, most *Liolaemus* species present taxonomic problems due to high levels of within species morphological and coloration variation (e.g., *Liolaemus nigromaculatus*, Troncoso-Palacios & Garin, 2013), or to the presence of convergence of morphological traits coupled with genetically divergent lineages (e.g., *Liolaemus monticola*, Torres-Pérez et al., 2009). This scenario has led to some subspecies being recently elevated to species (e.g., *L. nigromaculatus atacamensis*, now *L. atacamensis*; Simonetti et al., 1995; Valladares, 2011; Troncoso-Palacios & Garin, 2013), others becoming synonyms (e.g., *L. josephorum* under *L. velosoi*, Núñez, Schulte II & Garín, 2001; *L. lonquimayensis* under *L. elongatus*, Troncoso-Palacios et al., 2016a), and several candidate species proposed (e.g., Cianferoni et al., 2013; Troncoso-Palacios et al., 2015). Therefore, it is necessary to infer a reliable phylogeny to clarify the evolutionary relationships of the group in order to establish a straightforward taxonomy.

In this study, we aim to develop a preliminary yet reliable phylogenetic tree for lizards within the *Liolaemus* subgenus using a sample of representative species from the major groups, namely the *alticolor-bibronii*, *gravenhorstii*, *nigromaculatus*, *nigroviridis*, *pictus*, and *robertmertensi* groups (Table 1). Classification should reflect natural groups, therefore our main aim is to provide an initial phylogenomic hypothesis of the subgenus *Liolaemus*, and illustrate the potentials of genomic scale data in disentangling the phylogenetic relationships of this puzzling taxon. In addition we aim to test the monophyly of some of the species groups currently recognized.

This study differs from previous studies focused on the subgenus *Liolaemus*, which have mainly used different fragments of the mitochondrial genome (e.g., Schulte II et al., 2000; Torres-Pérez et al., 2009; Guerrero et al., 2013; Troncoso-Palacios et al., 2016a; Troncoso-Palacios et al., 2016b), in its large character sampling and analytical tools. Nevertheless, we are aware that incomplete taxon sampling has drawbacks, but the new phylogenomic tree presented here should be considered a reliable starting hypothesis based on a high volume of data. We use a sequence capture method, which employs short probes (60–120 base pairs) to hybridize to specific genomic regions, which are then isolated and sequenced using next-generation sequencing (Gnirke et al., 2009; McCormack et al., 2012), a method successfully used to resolve relationships among mammals (McCormack et al., 2012), birds (McCormack et al., 2013), turtles (Crawford et al., 2012) and squamates (Pyron et al., 2014; Leaché et al., 2016), among others. We use Maximum Likelihood as well as phylogenetic methods that have been developed to estimate species trees using the multispecies coalescent model to accommodate the stochastic segregation of multiple independent loci (Rannala & Yang, 2003). Coalescent approaches are advantageous for phylogenomics, because their accuracy increases as more loci are sampled (Edwards et al., 2016; Leaché & Rannala, 2011).

## Materials and Methods

### Sampling

We followed the classification of *Liolaemus* advanced by Abdala & Quinteros (2013) (Table 1), but updated it with recent species descriptions and taxonomic rearrangements (see Table S1 for details). We selected 16 taxa from the *alticolor-bibronii* (*L. fuscus* and *L. paulinae*), *gravenhorstii* (*L. cyanogaster*), *monticola* (*L. monticola*), *nigromaculatus* (*L. atacamensis, L. nigromaculatus, L. platei, L. velosoi*, and *L. zapallarensis*), *nigroviridis* (*L. isabelae* and *L. nigroviridis*)*, pictus* (*L. tenuis* and *L. pictus*) and *robertmertensi* (*L. nitidus*) species groups of the subgenus *Liolaemus* (Table 2). Within *Liolaemus tenuis*, two subspecies have been described (Müller & Hellmich, 1933) based primarily on color patterns: *Liolaemus t. tenuis* (*terra typica*: Santiago) and *L. t. punctatissimus* (*terra typica*: Lota). Nevertheless, few authors recognize these two taxa as their distributions partially overlap (Formas, 1979), conflicting with the subspecies concept (Mayr & Ashlock, 1991). A recent phylogeographic study found three deeply divergent clades within *Liolaemus tenuis sensu lato*, suggesting these could constitute distinct species (Muñoz-Mendoza et al., 2017). Therefore, we have included representatives of those three lineages in this study, representing the two subspecies of *Liolaemus tenuis* (*L. t. tenuis* and *L. t. punctatissimus*), as well as the third divergent lineage found within the nominal *Liolaemus tenuis*, which is here referred to as *L. sp.* When possible, we used specimens collected at type localities (Table 2); detailed information regarding collection localities, including GPS coordinates, are found in Table S2. Specimens included in this study are deposited in the collection of Museo de Zoología de la Universidad de Concepción (MZUC), in the Monte L. Bean Life Science Museum at Brigham Young University (BYU), in the Laboratory of Zoology, Epidemiology and Evolution of the Pontificia Universidad Católica de Valparaíso (PUCV) and in the Colección Patricio Sánchez Reyes de la Pontificia Universidad Católica de Chile (SSUC) (Table 2). Those specimens housed at MZUC were collected by us under collection permits No 9487/2014, 1898 and 4729 (SAG) and 04/2013 IX (CONAF) and were sacrificed by a pericardiac injection of sodium tiopenthal (Abbot^®^/Pentovet^®^).

**Table 2:** Specimens of *Liolaemus* included in the study. Species assignment; museum catalog number, collection locality data as well as distance to type locality is provided. Distances are given in kilometers (in straight line). Voucher abbreviations are as follows: BYU, Brigham Young University, Monte L. Bean Life Science Museum, Provo, Utah, United States; SSUC, Colección Patricio Sáchez Reyes de la Pontificia Universidad Católica de Chile, Santiago, Chile; MZUC, Museo de Zoología de la Universidad de Concepción, Concepción, Chile.

Species	Voucher	Collection locality	Type locality	Distance to type locality
*L. atacamensis*	MZUC-45084	Finca de Chañaral	Atacama, north of Copiapó	60
*L. cyanogaster*	MZUC-45092	San Pedro Station	Valdivia	45
*L. fuscus*	MZUC-45085	La Herradura, Coquimbo	Valparaíso	335
*L. isabelae*	MZUC-45086	Near Salar de Pedernales	Near Salar de Pedernales	20
*L. monticola*	LMON619^*^	El Yeso	San Francisco River	150
*L. nigromaculatus*	SSUC 643	Las Terrazas Beach, Paposo	Between Puerto Viejo and Copiapó	236
*L. nigroviridis*	LNIG614^*^	El Yeso	San Francisco River	150
*L. nitidus*	MZUC-45087	Algarrobo	Valparaíso	34
*L. paulinae*	MZUC-45088	Calama	Calama on the Loa River	0
*L. pictus*	MZUC-45094	Valdivia National Park	Valdivia	30
*L. platei*	MZUC-45089	Quebrada Buenos Aires	Coquimbo	43
*L. sp.*	BYU 49951	Caracol	–	–
*L. t. punctatissimus*	BYU 48375	Lota	Lota	0
*L. t. tenuis*	MZUC-45093	Til-Til	Santiago	32
*L. velosoi*	MZUC-45090	Nantoco	Detour Cerro Imán, close to Copiapó	35
*L. zapallarensis*	MZUC-45091	Quebrada Buenos Aires	Zapallar	330

**Notes.**

* individual deposited in the Zoology, Epidemiology and Evolution Laboratory at the Pontificia Universidad Católica de Valparaso, Valparaíso, Chile

### Library preparation, target enrichment, and sequencing

We extracted DNA using the DNeasy extraction kit (Qiagen, Inc., Hilden, Germany), and quantified DNA concentrations for each sample using a Qubit fluorometer (Life Technologies, Inc., Carlsbad, CA, USA). We randomly sheared 300 ng of DNA to a target size of approximately 400–600 bp by sonication with seven cycles using Bioruptor Pico (Diagenode BioRuptor; Diagenode, Liège, Belgium); fragment size distributions were verified using Agilient Tape Station 2200 (Agilient Tech.).

We prepared sequencing libraries using TruSeq Nano DNA Sample Prep Kit (http://www.illumina.com/products/truseq-nano-dna-sample-prep-kit.ilmn) following, with some modifications, the protocols available at http://ultraconserved.org/#protocols. We attached adapters from MyBaits custom probe kit (http://www.mycroarray.com/mybaits/mybaits-custom.html) to each library. We used probes designed by Leaché et al. (2015) specific for iguanian lizards, targeting 541 ultraconserved elements (herein UCEs) (which are a subset of the 5472 UCEs published by Faircloth et al., 2012) and 44 genes from the Squamate Tree of Life project (Wiens et al., 2012). UCEs are highly conserved regions (>60 base pairs) with flanks that exhibit increasing variation as the distance from the conserved core increases (Bejerano et al., 2004; Faircloth et al., 2012). After library amplification, we quantified 2 µL of each library using fluorometry (Qubit; Life Technologies, Carlsbad, CA, USA), and prepared two pools each containing eight libraries totaling 500 ng per pool (62.5 ng each library). We concentrated library pools using a Vacufuge Plus vacuum concentrator (Eppendorf, Hamburg, Germany) and rehydrated each library in 3.4 µL of ddH2O. We enriched pooled libraries using a synthesis of 1170 RNA probes (two 120 bp probes per locus) (Mycroarray, Inc., Ann Arbor, MI, USA). Libraries were incubated with RNA probes for 24 h at 65 °C. Post-hybridized libraries were enriched using TruSeq adapter primers with Phusion High-Fidelity DNA polymerase (New England BioLabs Inc) in order to minimize errors during PCR (Brelsford et al., 2012), for 17 cycles and cleaned by bead purification. We quantified enrichment by comparing non-enriched libraries versus enriched libraries in a qPCR (Applied Biosystems Inc., Foster City, CA, USA) with primers targeting five loci mapping to five chromosomes (based on the genome of *Anolis carolinensis,* Alföldi et al., 2011).

We sequenced the enrichment products on a single PE100 Illumina HiSeq2500 lane. Sequencing was performed at the QB3 Vincent J. Coates Sequencing Laboratory at the University of California, Berkeley.

### Sequence processing

Particular sequences were associated with individual specimens using strict matching of their unique bar codes in CASAVA (Illumina, Inc.). Demultiplexed sequence reads were subjected to quality control in Trimmomatic (Bolger, Lohse & Usadel, 2014), which removes contaminating adapter sequences and low quality bases. For this, we followed the analytical pipeline available at http://phyluce.readthedocs.io/en/latest/quality-control.html and used the parallel wrapper script Illumiprocessor (http://dx.doi.org/10.6079/J9ILL). Clean reads were assembled using the *de novo* assembler IDBA (Peng et al., 2012), following the pipeline available at: http://phyluce.readthedocs.io/en/latest/assembly.html. We repeated our IDBA assemblies iteratively over k-mer values from 50 to 90, and selected the k-mer value that produced the largest N50 value for each species. Although the majority of loci were sequenced across species, a significant proportion of loci were not recovered in *Liolaemus tenuis tenuis* (with only nine of the 585 loci captured). Therefore, instead of choosing the k-mer with the highest N50 value for *L. t. tenuis* (Table S3), we chose the k-mer that maximized the number of loci recovered (nine loci, k-mer = 50; Table 3). In addition, to test if missing data influenced tree topologies or support values, we inferred phylogenetic trees with matrices including and excluding *Liolaemus tenuis tenuis*.

**Table 3:** *De novo* assembly results from IDBA for *Liolaemus* sequence capture data. For each species, we report the selected k-mer value, number of contigs, N50 value, and number of sequenced loci (*N*) for protein-coding genes and UCEs in the final alignment.

Species	K-mer	Contigs	N50	*N*	Protein-coding	UCEs
*L. atacamensis*	90	949	388	549	37	511
*L. cyanogaster*	80	1,780	357	543	25	517
*L. fuscus*	90	1,044	326	558	37	520
*L. isabelae*	80	2,150	257	547	30	516
*L. monticola*	90	2,803	331	464	34	429
*L. nigromaculatus*	90	428	286	216	16	199
*L. nigroviridis*	90	253,864	111	424	29	394
*L. nitidus*	90	272,920	112	496	37	458
*L. paulinae*	80	111,191	320	503	33	468
*L. pictus*	90	574	331	360	22	337
*L. platei*	80	2,160	257	465	29	435
*L. sp.*	90	144	327	138	8	130
*L. t. punctatissimus*	90	517	307	328	24	304
*L. t. tenuis*	50	235	133	9	2	7
*L. velosoi*	80	57,514	293	480	25	453
*L. zapallarensis*	80	27,903	239	502	33	468

We used phyluce (Faircloth, 2015) to assemble loci across species and to produce alignments for phylogenetic analysis. We started by aligning species-specific contigs to the set of probes (match contigs to probes.py) with LASTZ (Harris, 2007). We aligned FASTA sequences of each locus using MAFFT (Katoh & Standley, 2013) using the phyluce_align_seqcap_align command. Sequence capture raw reads are available at NCBI (SRA SRP106329). Sequence alignments and phylogenetic trees are available from Dryad doi: 10.5061/dryad.35q4h.

### Phylogenetic analyses

Because it has been suggested that the use of gene trees based on relatively low variation markers could be problematic for species tree analyses (Lanier, Huang & Knowles, 2014; Xi, Liu & Davis, 2015; Xu & Yang, 2016), and some empirical studies using UCE data have supported this claim (e.g., McCormack et al., 2013), we conducted phylogenetic analyses using three different data sets: (1) ultraconserved elements (538 loci); (2) protein-coding genes (41 loci), and (3) a combined data set containing all UCEs and protein-coding genes (581 loci). Our comparison of phylogenetic trees estimated with UCEs and protein-coding genes allowed us to explore the phylogenetic signal in the different types of markers and make inferences about their utility for phylogenetic inference. Moreover, we estimated phylogenetic trees using three different approaches: (1) Maximum Likelihood, (2) quartet inference, and (3) a gene-tree summary method. Based on the phylogenies inferred by Schulte II et al. (2000) and Pyron, Burbrink & Wiens (2013), we used sequences of *Liolaemus cyanogaster* to root the trees, as this species appears in a clade (along with *L. pictus*) that is sister to a clade containing the rest of the species included in this analyses.

Before phylogenetic inference, summaries of the sequence capture loci were generated using scripts available from https://github.com/dportik/Alignment_Assessment (Portik, Smith & Bi, 2016). These, in addition to visual inspection of individual gene trees, allowed us to detect outliers that could result from poor alignments. These loci were manually edited and checked again using the same pipeline.

#### Maximum likelihood

We inferred a Maximum Likelihood tree with IQ-TREE 1.5.5. (Nguyen et al., 2015). Concatenated sequences for the three data sets (with partition information) were generated using PhD_Easy.py available at https://github.com/ODiogoSilva/ElConcatenero3. Gene-partitioned alignments were analyzed, and the model that best fits the data was determined by IQ-TREE according to the Bayesian information criterion (BIC) (Chernomor, Haeseler & Minh, 2016; Kalyaanamoorthy et al., 2017). The FreeRate heterogeneity model, which infers the site rates directly from the data, was implemented (Soubrier et al., 2012). To assess branch support, we used the ultrafast bootstrap approximation (UFboot) with 1,000 replicates (Minh, Nguyen & Von Haeseler, 2013).

#### Quartet inference

We used SVD quartets (Chifman & Kubatko, 2014) as implemented in PAUP* (Swofford, 2002) to infer a species tree. This method, unlike gene tree summary statistics methods, uses sequence data directly and therefore incorporates more sources of variability in the species tree estimation process (Chifman & Kubatko, 2014). We evaluated all possible quartets and treated ambiguities as missing data. We conducted 1,000 bootstrap (BS) replicates.

#### Gene tree-based analyses

These methods use rooted (e.g., MP-EST, Liu, Yu & Edwards, 2010) or unrooted gene trees (e.g., ASTRAL-II, Mirarab et al., 2014) as an input to generate a species tree. To estimate the appropriate substitution model for each locus, we used modeltest_runner.py (available at https://github.com/cwlinkem/linkuce), a wrapper script to run jmodeltest (Posada, 2008), and the Bayesian information criterion (BIC; Schwarz, 1978). Next, we estimated ML trees using RAxML v8.2 (Stamatakis, 2014). ML analyses used the models selected from jmodeltest, and implemented an auto-bootstrap procedure (Pattengale et al., 2010). To infer the species tree we used ASTRAL-II (Mirarab et al., 2014), which can outperform other methods when levels of incomplete lineage sorting are high (Chou et al., 2015). One advantage of ASTRAL-II is that it does not need rooted trees (the outgroup was not amplified for all loci used). In addition, ASTRAL-II has been shown to perform well using UCEs in difficult phylogenetic scenarios (Meiklejohn et al., 2016). To generate the species tree, we input ML bootstrap trees of all loci into ASTRAL-II and performed 1,000 bootstrap (BS) replicates, by sampling with replacement from the ML bootstrap gene tree files.

## Results

### Sequence capture data

The number of loci (UCEs and protein-coding genes) recovered per species is shown in Table 3. Our final incomplete dataset yielded a total of 581 loci, which consisted of 538 UCEs and 43 protein-coding genes. Four loci were only amplified for two taxa, so they were automatically excluded from the final dataset. These loci consisted in three UCEs (corresponding to chromosomes z_3515 and z_5971, and chromosome 9_7188) and one protein-coding gene (UBN1: ubinuclein1). Only nine loci were successfully amplified for *Liolaemus tenuis tenuis*; of these, only one was shared across all 16 terminals. If *L. t. tenuis* is removed, the total number of shared loci across the remaining 15 terminals rises to 20.

**Figure 1:**
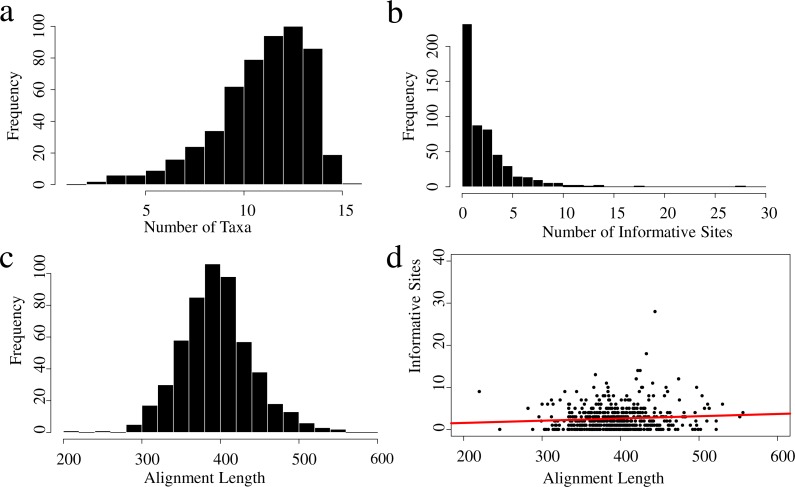
Properties of the UCEs data set for the 16 taxa used in this study. Frequency distributions show the (A) number of taxa across alignments; (B) number of informative sites per locus; (C) alignment length distributions and (D) informative sites per alignment length, the red line indicates the adjusted *R*^2^ = 0.005577 (*p* = 0.04569).

**Figure 2:**
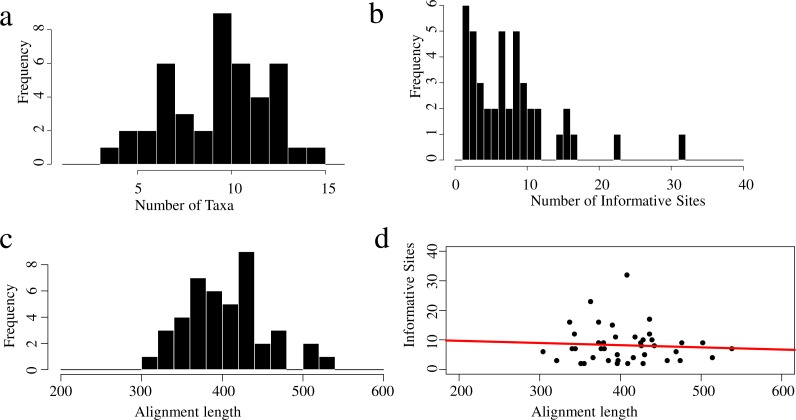
Properties of the protein-coding gene data set for the 16 taxa used in this study. Frequency distributions show the (A) number of taxa across alignments; (B) number of informative sites per locus; (C) alignment length distributions and (D) informative sites per alignment length, the red line indicates the adjusted *R*^2^ =  − 0.02026 (*p* = 0.6857).

**Figure 3:**
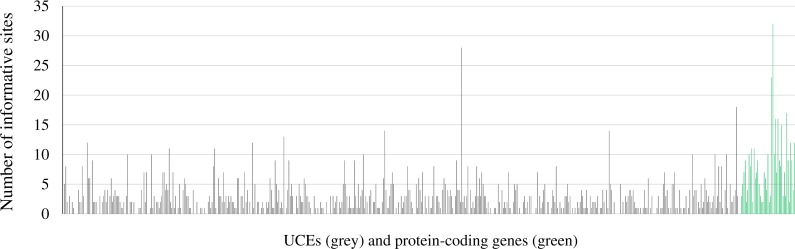
Number of informative sites per marker. Bars in grey correspond to ultraconserved elements and green bars to protein-coding genes.

Summaries of the sequence capture loci are available in Figs. 1 and 2. The average number of base-pairs across alignments was 396, and both average percent of gaps per alignment and average percent of missing data across alignments were 0.4%. Protein-coding genes were more informative than UCEs, with 2.1% and 0.7% of informative sites, respectively (Table 4). Numbers of informative sites per marker are shown in Fig. 3 and Table S4. The substitution models inferred for all loci are available at Table S5. The best-fitting model most often identified by jmodeltest was F81 (46% of markers), followed by HKY (21%) and JC (13%).

### Phylogenetic analyses

**Figure 4:**
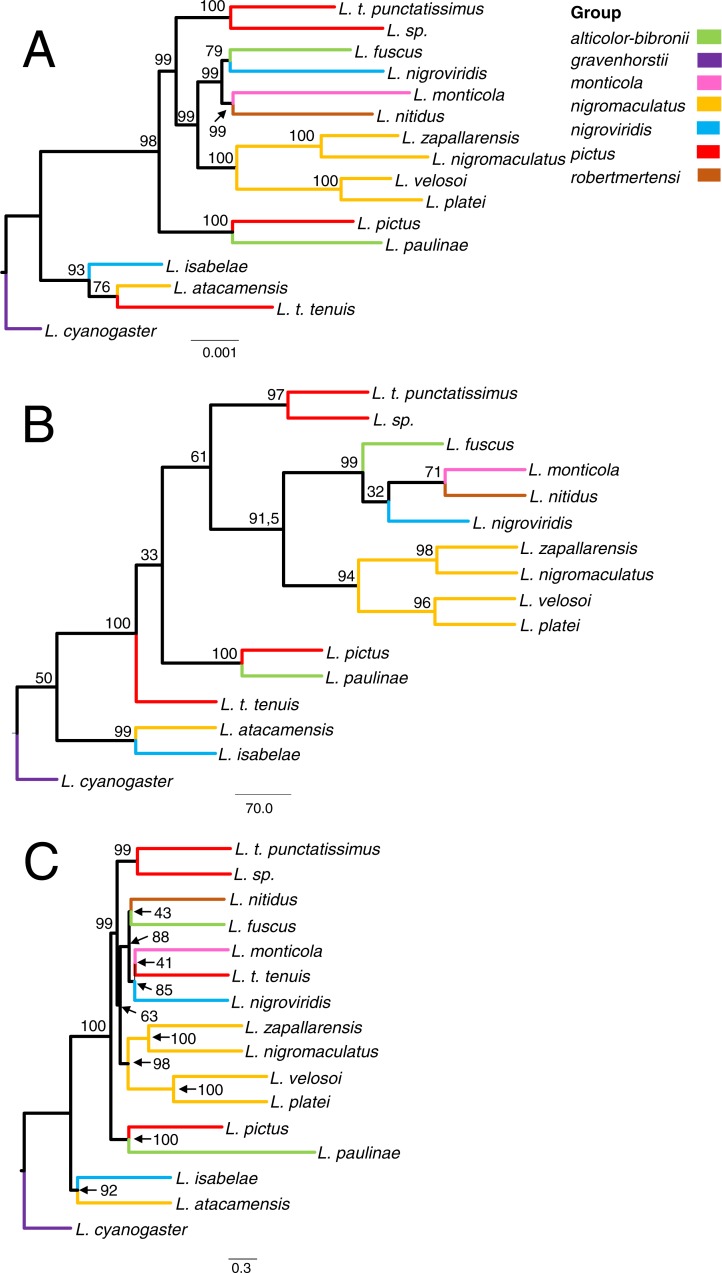
Phylogenomic relationships among *Liolaemus* lizards from the Chilean groups, estimated with sequence capture data (protein-coding genes + UCEs) using maximum likelihood (A), quartet based (B) and gene-tree based methods (C). The assignation of taxa to species group was done following Abdala & Quinteros (2013). Branch color indicates assignation of species to species groups according to the classification specified in the figure legend. Values next to nodes indicate bootstrap support.

**Figure 5:**
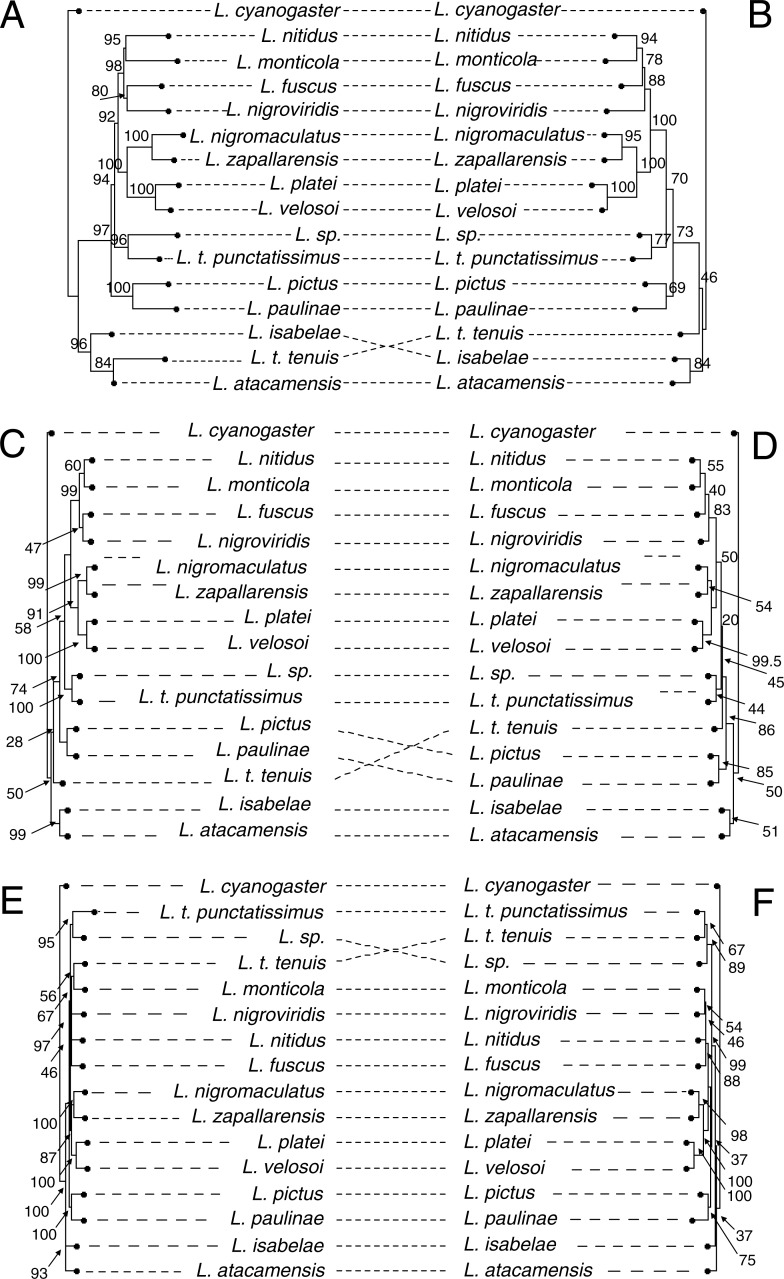
Mirror images of the phylogenies inferred using IQ-TREE (A: ultra-conserved elements; B: protein-coding genes), SVD quartets (C: ultra-conserved elements; D: protein-coding genes), and ASTRAL-II (E: ultra-conserved elements; F: protein-coding genes). Values above nodes indicate bootstrap support.

Most analyses support the same topology in which *L. fuscus*, *L. monticola*, *L. nigroviridis,* and *L. nitidus* always formed a highly supported clade, which in turn is sister to another well-supported clade formed by *L. nigromaculatus*, *L. platei*, *L. velosoi* and *L. zapallarensis* (Figs. 4 and 5). In most cases, this group of species is sister to a clade formed by *L. t. punctatissimus* and *L. sp.* This whole clade is sister to a clade formed by *L. pictus* and *L. paulinae*. Sister to this grouping is a clade containing *L. atacamensis* and *L. isabelae*. Although most analyses inferred these relationships, the only clades that received strong support across all analyses (BS ≥ 70%) are the following: *L. fuscus*, *L. monticola*, *L. nigroviridis*, and *L. nitidus,* and *L. platei* sister to *L. velosoi* (Fig. 5). The same general relationships among species, as well as highly similar bootstrap support values, are retrieved when phylogenetic trees are inferred excluding *Liolaemus tenuis tenuis* (Figs. S1–S3). The only clade that exhibits a considerable difference in bootstrap support in comparison to the 16-taxa phylogeny is the one inferred with SVD quartets (Clade I, Table S6) that includes all species but *Liolaemus atacamensis* and *L. isabelae*.

**Table 4:** Features of the UCEs and protein-coding genes.

Marker type	*N*	Average length	Average # taxa	Average % informative sites	Average % gaps and missing data
UCE	538	395.5	11.4	0.7	0.5
Protein-coding	43	406.3	9.8	2.1	0.2

Other relationships received high support in all analyses except when phylogenies were inferred using only the protein-coding genes matrix in IQ-TREE (*L. pictus* sister to *L. paulinae*, BS = 69), in SVD quartets (*L. atacamensis* sister to *L. isabelae*, BS = 51; *L. t. punctatissimus* sister to *L. sp.*, BS = 44; *L. zapallarensis* sister to *L. nigromaculatus*, BS = 54; and the clade formed by *L. nigromaculatus*, *L. zapallarensis*, *L. platei* and *L. velosoi*, BS = 49) (Fig. 5). Overall, bootstrap support values were higher for the phylogenies inferred with UCEs and for the combined data set than for those inferred with only protein-coding genes. Moreover, some differences were observed in some of the analyses (described below). Remarkably, all species groups from which more than one species were sampled are polyphyletic (Fig. 4).

#### Maximum likelihood

Substitution models (as retrieved by IQ-TREE) for each partition can be found in Table S5. The ML analysis of the combined data supports a clade (BS = 93) containing *L. isabelae*, *L. atacamensis* and *L. t. tenuis* as sister to a clade formed by the remaining species (BS = 98); within the former clade *L. atacamensis* and *L. t. tenuis* are sister to each other (BS = 76) (Fig. 4A). Meanwhile, within the second main clade of *Liolaemus*, *L. paulinae* and *L. pictus* are sister species (BS = 100), and this clade is sister to the remaining species of the clade (BS = 98). *Liolaemus t. punctatissimus* and *L. sp.* are sister to each other (BS = 100) and together, sister to a clade (BS = 99) containing two smaller clades, each composed of two subclades: one (BS = 99) contains (*L. fuscus* + *L. nigroviridis*) and (*L. monticola* + *L. nitidus*), while the other (BS = 100) contains (*L. zapallarensis* + *L. nigromaculatus*) and (*L. velosoi* + *L. platei*).

There are several key differences between the protein-coding vs. UCE-based topologies (Figs. 5A, 5B). First, the bootstrap support values for the protein-coding tree are generally lower than those for the UCE tree (Figs. 5A, 5B). Second, the protein-coding tree fails to support the *L. fuscus* + *L. nigroviridis* clade. Third, the protein-coding tree places *L. t. tenuis* as sister to the clade composed by the remaining species while the UCE tree retrieves a sister species relationship between *L. t. tenuis* and *L. atacamensis*.

#### Quartet inference

In SVD quartets, 1820 quartets were analyzed, and the ratio of incompatible to compatible quartets was 240/1104 for the UCEs (21.7%; *N* = 1,344 informative quartets), 157/649 for the protein-coding genes (2.3%; *N* = 806), and 227/1132 for the combined data (20.0%; *N* = 1,359). Overall, SVD quartets inferred the same relationships as maximum likelihood (Figs. 4 and 5). Again, the main differences relative to the tree resulting from the IQ-TREE phylogeny (full data set) are the relationships between *L. monticola. L. nitidus, L. fuscus* and *L. nigroviridis*, as well as the placement of *L. t. tenuis* (Fig. 4)*.* As opposed to its placement in Fig. 4A, in SVD quartets *L. t. tenuis* is not sister to *L. atacamensis* but diverges right after (*L. atacamensis* + *L. isabelae*) (Fig. 4B). In the combined tree (Fig. 4B) the topology shows the following arrangement (*L. fuscus* (*L. nigroviridis* (*L. monticola*, *L. nitidus*))) (BS = 99.4), while in the protein-coding tree (Fig. 5D) relationships are (*L. nigroviridis* (*L. fuscus* (*L. monticola*, *L. nitidus*))) (BS = 82.9). In the combined tree as well as in the UCE tree (Figs. 4 and 5C), *L. atacamensis* and *L. isabelae* form a clade that is sister to that formed by all other species; within the later *L. t. tenuis* is sister to the other species. Meanwhile, in the protein coding tree *L. atacamensis* and *L. isabelae* are also sister to all other species (BS = 50), but the clade formed by *L. paulinae* + *L. pictus* is sister to the remaining species. As in the IQ-TREE phylogenies, overall bootstrap support values of trees estimates with protein-coding genes seem to be lower than UCE-based trees (Figs. 5C, 5D).

#### Gene-tree summary method

Overall, ASTRAL-II inferred the same relationships as the Maximum Likelihood phylogeny with the full data set (Figs. 4A, 4C). As in other cases, the placement of *L. t. tenuis* in the phylogeny varies when different data sets are used. When phylogenies are inferred with the full data set and the UCE data set, *L. t. tenuis* is sister to *L. monticola* (BS = 41 and BS = 56, respectively; Figs. 4C and 5E), while the protein-coding tree (Fig. 5F) supports *L. t. tenuis* as sister to *L. t. punctatissimus* (BS = 67). The protein-coding tree also supports a (*L. fuscus* (*L. nitidus* (*L. monticola*, *L. nigroviridis*))) clade (BS = 88), but does not support the clade of *L. atacamensis* and *L. isabelae.* Nevertheless, the key difference is that, considering all inferred phylogenies, only in the tree inferred with ASTRAL-II using protein-coding genes, the clades composed by *L. fuscus*, *L. monticola*, *L. nigroviridis* and *L. tenuis* + *L. nigromaculatus*, *L. zapallarensis*, *L. platei*, and *L. velosoi* are not sister to *L. t. punctatissimus* and *L. sp*.; they are more closely related to the *L. pictus* +* L. paulinae* clade. Also in ASTRAL-II, support values are lower for the protein-coding tree relative to the UCE-tree.

## Discussion

Studies of reptile systematics are starting to make the transition to a phylogenomic approach (Leaché et al., 2016; Grismer et al., 2016; Streicher & Wiens, 2016). Until now, phylogenetic studies of liolaemid lizards have been based on a single locus, or a few nuclear loci (Schulte II et al., 2000; Troncoso-Palacios et al., 2015; but see Avila et al., 2015; Olave et al., 2015). Including multiple loci in phylogenetics is advantageous, because it helps attain the statistical power that is typically necessary for resolving discordances that arise among independent loci (Edwards, 2009). This is the first study applying phylogenomic data to generate a genome-scale phylogeny for *Liolaemus*. We emphasize that these analyses do not represent an attempt to assess the benefits of using different data types; the goal of this study was rather to infer a phylogeny that would serve as a starting hypothesis of the evolutionary relationships of lizards of the *Liolaemus* subgenus.

The relatively poor understanding of the evolutionary history of *Liolaemus* has its root in many factors, including phylogenetic analyses with incomplete taxon sampling, the use of few markers in molecular-based studies, and the discordance between morphological and DNA-based groupings (e.g., Lobo, 2005 vs. Schulte II et al., 2000). In this study, we focused on the subgenus *Liolaemus* by studying a sample of species allocated to its two sections, the *nigromaculatus* and *chiliensis* sections, and to seven species groups (*sensu* Abdala & Quinteros, 2013; Troncoso-Palacios et al., 2015; see Table 1). Despite the fact that our sample of species is modest relative to the real species richness of the subgenus, our sampling is still adequate for demonstrating that several sections and species groups are not monophyletic. Nevertheless, our results should be interpreted with caution; if bias in phylogenetic inference exists (such as base composition convergence, long branch attraction, among others) phylogenetic analyses may converge in an incorrect tree with higher support as new data is added (Swofford et al., 2001). Therefore, increased taxon sampling would be a way to help overcome these biases should they exist (Reddy et al., 2017).

We conducted a comparison of the UCEs and protein-coding genes to determine the utility of different types of markers on phylogenetic inference for our particular data set, as it has been shown that data type may influence in phylogenetic inference (Reddy et al., 2017). Studies that have assessed the efficacy of UCEs have suggested that they can provide high levels of phylogenetic informativeness (Blaimer et al., 2015). Overall, our results indicate that bootstrap values are generally higher when using ∼540 UCEs versus ∼40 protein-coding genes. However, on average, the protein-coding genes contained more variation (2.1% versus 0.7% variation) compared to UCEs (Table 4). Many of the UCEs had few or no informative sites (Fig. 1). This is not surprising as UCEs are not expected to exhibit high variation at the core region, but variation increases based on distance from the center of the UCE (Faircloth et al., 2012). As our UCE assembled loci were relatively short (<400 bp; Table 4), they do not show much variation. However, the UCEs outnumbered the protein-coding genes by 10-fold. Therefore, this problem of low-information content in individual loci can be partially alleviated by sampling more loci (Xi, Liu & Davis, 2015). This is in agreement with the findings of Blom et al. (2016); in their study, the inclusion of low resolution gene trees (inferred from relatively uninformative loci) increased the consistency of phylogenetic inference with higher average BS values. This contrasts with findings by other authors in which using uninformative loci reduced the accuracy of species tree estimation (e.g., Xi, Liu & Davis, 2015). Nevertheless, they too conclude that sampling more loci can alleviate this issue, even if they also show low information content.

Overall, and not considering *Liolaemus tenuis tenuis* position in the phylogeny, the subsets of the data that we analyzed (UCEs and protein-coding genes, and the combined data) supported the same phylogeny regardless of the analytical approach (concatenation versus coalescent methods). The only inconsistency in topology appeared in the protein-coding tree inferred with ASTRAL-II, where the clade containing *L. platei*, *L. velosoi*, *L. nigromaculatus*, *L. zapallarensis*, *L. monticola*, *L. nitidus*, *L. fuscus* and *L. nigroviridis* appeared more closely related to *L. pictus* + *L. paulinae* than to *L. t. punctatissimus* + *L. sp.* (Fig. 5); nevertheless, this relationship was not strongly supported (BS = 37). On the other hand, some aspects of the phylogeny remain unresolved; for example, the relationships between *L. fuscus, L. monticola, L. nitidus and L. nigroviridis*. These four species form a well-supported clade (Fig. 5), but relationships among them are unclear. It was earlier suggested that *Liolaemus* underwent a rapid radiation (Olave et al., 2015; Pincheira-Donoso, Harvey & Ruta, 2015) and this could cause some relationships to be difficult to resolve; further studies with an even denser taxon or character sampling may prove useful to fully resolve these relationships, or perhaps they represent a hard polytomy (Suh, 2016; Olave et al., 2016).

Below, we briefly describe our findings within each of the sampled species groups that contained at least two sampled species. Despite the fact that these inferences are based on a small number of species, they are representative of several groups within *Liolaemus* and they are thus a starting point from which to keep building a robust phylogeny and at the end, a natural classification of *Liolaemus*.

The *chiliensis* section: the *alticolor-bibronii* species group (Espinoza, Wiens & Tracy, 2004; Lobo, 2005; Lobo, Espinoza & Quinteros, 2010; Quinteros, 2012). This group is represented by species occurring mainly along the Andes in Argentina, Bolivia, Chile, and Peru (Quinteros, 2012). There have been frequent changes in the taxonomic composition of this group; initially, it contained 10 species (Espinoza, Wiens & Tracy, 2004), a number that has grown to 26 species (Lobo, 2005: 12 species; Lobo, Espinoza & Quinteros, 2010: 22 species; Quinteros, 2012: 24 species) (Table S1). Our results show that the *alticolor-bibronii* species group is polyphyletic, as *L. paulinae* and *L. fuscus* are not sister to each other. *Liolaemus paulinae* is supported as sister of *L. pictus* (see Figs. 4 and 5). It needs to be considered that many species of this group were not included in the analyses, and as such the clade formed by these geographically distant species, *L. paulinae* from desert areas of northern Chile, and *L. pictus* from austral *Notophagus* forests (Pincheira-Donoso & Núñez, 2005), may be the result of this incomplete taxonomic coverage. On the other hand, *L. fuscus* is consistently recovered in a clade containing also *L. monticola*, *L. nigroviridis* and *L. nitidus*, a relationship that has been previously suggested in earlier studies with mitochondrial markers (e.g., Schulte II et al., 2000) and is further confirmed here.

The *chiliensis* section: the *pictus* species group (Cei, 1986). This group, as currently defined, includes species from Argentina and Chile. Of its five species, we have included two: *L. pictus* and *L. tenuis.* The latter was represented by three forms that could represent distinct species (Muñoz-Mendoza et al., 2017). As mentioned before for the *alticolor-bibronii* group, *L. pictus* is consistently placed and with strong support as sister to *L. paulinae*. Therefore, *L. pictus*, *L. t. tenuis*, *L. t. punctatissimus* and *L. sp.* fail to form a monophyletic group; the *pictus* group is then retrieved as polyphyletic in our analyses (Fig. 4). The position of *Liolaemus tenuis s. s.* within the radiation of *Liolaemus* remains unresolved, as it changes according to the data set and approach used (see Figs. 4 and 5). Only once, in the species tree inferred using ASTRAL-II with protein-coding genes, *L. t. tenuis* was recovered as related to its two presumably closely related forms, *L*. *t. punctatissimus* and *L. sp.* (Fig. 5), which in turn are retrieved as well- supported sister species in every analysis. However, it should be noted that the data generated for *Liolaemus tenuis s. s.* is minimal, consisting of only two protein-coding genes and seven UCEs. As such, the phylogenetic position of *L. t. tenuis* remains unresolved, awaiting the generation and analysis of additional data.

The *nigromaculatus* section: the *nigromaculatus* species group. This species group has a complex taxonomic history. To our knowledge, there is only one DNA-based phylogenetic study focused on the group, which is based on mitochondrial DNA (Troncoso-Palacios et al., 2015). Most of the species from the *nigromaculatus* species group included in our study occur in sympatry. *Liolaemus nigromaculatus* and *L. velosoi* are found in the Atacama Region (Troncoso-Palacios & Garin, 2013; Troncoso-Palacios, 2014), while *L. platei* and *L. atacamensis* are present in the Atacama and Coquimbo Regions (Pincheira-Donoso & Núñez, 2005), and *L. zapallarensis* occurs in the Valparaíso and Coquimbo Regions (Pincheira-Donoso & Núñez, 2005). *Liolaemus atacamensis* and *L. zapallarensis* were first described as subspecies of *L. nigromaculatus* (Müller & Hellmich, 1933). In our analyses, four out of the five species from the *nigromaculatus* group analyzed always form a well-supported clade (Fig. 4). *Liolaemus atacamensis*, which does not group with either *L. zapallarensis* or *L. nigromaculatus*, appears as sister to *L. isabelae*, far from the other species of the *nigromaculatus* species group. Therefore, although we recovered most of the species of this group forming a clade, the *nigromaculatus* species group is polyphyletic. This result is unexpected since previous analyses based on mitochondrial DNA have always found that *L. atacamensis* and *L. nigromaculatus* are sister species, and morphologically, these species are highly similar (Troncoso-Palacios & Garin, 2013). Nevertheless, is not unlikely that inferences based on a single marker of mitochondrial marker versus >500 nuclear markers support a different tree (e.g., Moore, 1995). Troncoso-Palacios et al. (2015) recovered a *nigromaculatus* species group with strong support (posterior probability = 0.99), but *L. atacamensis* appeared as sister to *L. nigromaculatus.* This clade is sister to a clade comprising *L. isabelae*, *L. pseudolemniscatus* (not included in this study) and species from the “*platei* group” (*L. velosoi* + *L. platei*). In our study, *L. zapallarensis* and *L. nigromaculatus* are sister species, which are in turn sister of the aforementioned grouping (which is consistent with previous studies; e.g., Troncoso-Palacios & Garin, 2013). Nevertheless, and as previously mentioned, *L. atacamensis* is supported as sister to *L. isabelae* and not closely related to the “*platei*” group. In summary, our results support the *nigromaculatus* group if *L. atacamensis* is excluded from this group.

The *nigromaculatus* section: the *nigroviridis* species group. Species of the *nigroviridis* group are distributed in the highlands of central and northern Chile (Pincheira-Donoso & Núñez, 2005). Most species have a complex taxonomic history (a detailed summary can be found in Troncoso-Palacios et al., 2016a), which has been difficult to untangle due to the lack of genetic sampling. Our trees show that this species group is polyphyletic, as *L. isabelae* and *L. nigroviridis* are not sister species; *L. isabelae* is in the majority of the topologies sister to *L. atacamensis*, while *L. nigroviridis* is recovered consistently in a clade comprising *L. monticola*, *L. fuscus* and *L. nitidus.* These four species overlap in their distributions in the Chilean Central Valley. The most widespread species are *L. fuscus* (*alticolor-bibronii* group) and *L. nitidus* (*robertmertensi* group), which can be found from the Atacama Region (∼27°S) south to the Biobío Region (∼36°S) (Troncoso-Palacios, 2014). *Liolaemus nigroviridis* and *L. monticola* (*monticola* group) have a more restricted distribution, as the former species occurs in central Chile between latitudes ca. 30°to 34°S and above 1,100 m, in both the Andean and Coastal mountains (Cianferoni et al., 2013 and references therein), while the latter is found in the San Francisco valley (32°22′S, 70°25′W) at 1,700 m and also in coastal and transversal mountain ranges (33°S) between 600 and 1,800 m in central Chile (Torres-Pérez et al., 2009 and references therein). The relationships among these four species are not fully resolved; in most cases, *L. monticola* and *L. nitidus* appear to be sister species, while, as suggested by previous studies (Schulte II et al., 2000; Schulte & Moreno-Roark, 2010; Guerrero et al., 2013; Pyron, Burbrink & Wiens, 2013; Schulte, 2013; Troncoso-Palacios et al., 2015), *L. fuscus* and *L. nigroviridis* are sister to each other. Nevertheless, as the position among these four species varies with the dataset and method used (see Figs. 4 and 5), we cannot draw strong conclusions from these results.

Many authors, based on morphological data, have placed *L. isabelae* in the *nigroviridis* group (Lobo, 2005; Pincheira-Donoso & Núñez, 2005; Lobo, Espinoza & Quinteros, 2010), while previous DNA-based analyses do not support this placement, questioning the affiliation of *L. isabelae* to the *nigroviridis* group (Schulte & Moreno-Roark, 2010; Troncoso-Palacios et al., 2016a). Therefore, our phylogenomic study supports previous DNA based studies, suggesting a large amount of convergence in morphological character states between *L. isabelae* and *L. nigroviridis*. It is of interest to see if the suggested convergence is due to chance or promoted by living in similar highland habitats.

Regarding relationships among species groups, results also supports a sister relationship between the *monticola* and *nigroviridis* groups, and of this clade with the *nigromaculatus* group. Nevertheless, these results could change as species from other groups are included in the analysis.

## Conclusions

Among the main challenges of *Liolaemus* systematics is that all studies have partial taxonomic coverage. Our phylogenomic scale study failed to support the monophyly of most species groups, suggesting that morphological convergence has hampered an adequate classification in liolaemid lizards and that a reappraisal of groupings is necessary. A DNA-based study with broader taxonomic coverage should help redefine natural species groupings.

##  Supplemental Information

10.7717/peerj.3941/supp-1 Table S1 List of recent taxonomic work on the Chilean clade of *Liolaemus* Diversity of the Chilean clade of *Liolaemus* (species and groups) adapted and updated from Abdala & Quinteros (2013). Synonyms and additions relative to the classification proposed by Abdala & Quinteros (2013) are shown. The recently described *Liolaemus scorialis* Troncoso-Palacios et al. (2015) is *incertae sedis* in the *elongatus-kriegi* complex (Troncoso-Palacios et al., 2015). Species included in this study are marked in bold.

10.7717/peerj.3941/supp-2 Table S2 Detailed collection locality information Detailed locality data for the Chilean *Liolaemus* species included in the study. Coordinates are given in decimal degrees. Detailed type locality information, including coordinates when available, as well as bibliographic references are given.

10.7717/peerj.3941/supp-3 Table S3 Sequence capture assembly comparison. Comparison of *de novo* assembly results from IDBA across different k-mer values. The k-mer value with the highest N50 was chosen for each species (shown in bold). One exception is *L. t. tenuis*, which had too few loci.

10.7717/peerj.3941/supp-4 Table S4 Number of informative sites percentage of informative sites per marker

10.7717/peerj.3941/supp-5 Table S5 Models selected by the BIC criterion in IQ-TREE and jmodeltest for our sequence capture data set

10.7717/peerj.3941/supp-6 Table S6 Comparison of bootstrap support values estimated with phylogenies including and excluding *Liolamus tenuis tenuis* for the full data set (UCEs + protein-coding genes) Bold numbers indicate high bootstrap support values (>70) and asterisks indicate that *L. t. tenuis* belong to that particular clade.

10.7717/peerj.3941/supp-7 Figure S1 Phylogenomic relationships inferred for our sample of *Liolaemus* lizards excluding *Liolaemus tenuis tenuis,* with sequence capture data (protein-coding genes + UCEs) using maximum likelihood implemented in IQ-TREE Values next to nodes indicate bootstrap support.

10.7717/peerj.3941/supp-8 Figure S2 Phylogenomic relationships inferred for our sample of *Liolaemus* lizards excluding *Liolaemus tenuis tenuis,* with sequence capture data (protein-coding genes + UCEs) using a quartet-based method implemented in SVD quartets Values next to nodes indicate bootstrap support.

10.7717/peerj.3941/supp-9 Figure S3 Phylogenomic relationships inferred for our sample of *Liolaemus* lizards excluding *Liolaemus tenuis tenuis,* with sequence capture data (protein-coding genes + UCEs) using a gene-tree based method implemented in ASTRAL-II Values next to nodes indicate bootstrap support.

## Additional Information and Declarations

### DNA Deposition

The following information was supplied regarding the deposition of DNA sequences: Dryad: doi: 10.5061/dryad.35q4h.

### Data Availability

The following information was supplied regarding data availability: Our raw data is deposited at the NCBI SRA database. The accessions for our submission are: STUDY: PRJNA384329 (SRP106329) SAMPLE: Liolaemus_zapallarensis (SRS2163596) EXPERIMENT: L_zap (SRX2780506) RUN: Liolaemus_zapallarensis-READ1.fastq (SRR5501053) SAMPLE: Liolaemus_velosoi (SRS2163597) EXPERIMENT: L_vel (SRX2780507) RUN: Liolaemus_velosoi-READ1.fastq (SRR5501054) SAMPLE: Liolaemus_tenuis (SRS2163598) EXPERIMENT: L_ten (SRX2780508) RUN: Liolaemus_tenuis-READ1.fastq (SRR5501055) SAMPLE: Liolaemus_sp (SRS2163599) EXPERIMENT: L_sp (SRX2780509) RUN: Liolaemus_sp_nov-READ1.fastq (SRR5501056) SAMPLE: Liolaemus_tenuis_punctatissimus (SRS2163600) EXPERIMENT: L_ten_punc (SRX2780510) RUN: Liolaemus_punctatissimus-READ1.fastq (SRR5501057) SAMPLE: Liolaemus_platei (SRS2163601) EXPERIMENT: L_plat (SRX2780511) RUN: Liolaemus_platei-READ2.fastq (SRR5501058) SAMPLE: Liolaemus_pictus (SRS2163602) EXPERIMENT: L_pic (SRX2780512) RUN: Liolaemus_pictus-READ2.fastq (SRR5501059) SAMPLE: Liolaemus_paulinae (SRS2163603) EXPERIMENT: L_pau (SRX2780513) RUN: Liolaemus_paulinae-READ1.fastq (SRR5501060) SAMPLE: Liolaemus_nitidus (SRS2163604) EXPERIMENT: L_nit (SRX2780514) RUN: Liolaemus_nitidus-READ1.fastq (SRR5501061) SAMPLE: Liolaemus_nigroviridis (SRS2163605) EXPERIMENT: L_nigrov (SRX2780515) RUN: Liolaemus_nigroviridis-READ2.fastq (SRR5501062) SAMPLE: Liolaemus_nigromaculatus (SRS2163606) EXPERIMENT: L_nigrom (SRX2780516) RUN: Liolaemus_nigromaculatus-READ1.fastq (SRR5501063) SAMPLE: Liolaemus_monticola (SRS2163607) EXPERIMENT: L_mont (SRX2780517) RUN: Liolaemus_monticola-READ2.fastq (SRR5501064) SAMPLE: Liolaemus_isabelae (SRS2163608) EXPERIMENT: L_isab (SRX2780518) RUN: Liolaemus_isabelae-READ2.fastq (SRR5501065) SAMPLE: Liolaemus_fuscus (SRS2163609) EXPERIMENT: L_fusc (SRX2780519) RUN: Liolaemus_fuscus-READ2.fastq (SRR5501066) SAMPLE: Liolaemus_cyanogaster (SRS2163610) EXPERIMENT: L_cyan (SRX2780520) RUN: Liolaemus_cyanogaster-READ1.fastq (SRR5501067) SAMPLE: Liolaemus_atacamensis (SRS2163611) EXPERIMENT: L_atac (SRX2780521) RUN: Liolaemus_atacamensis-READ1.fastq (SRR5501068).
