# Supplementary material for: Phylogenomic analysis of the Chilean clade of Liolaemus lizards (Squamata: Liolaemidae) based on sequence capture data
Source: PeerJ. 2017 Oct 26;5:e3941. doi: 10.7717/peerj.3941 (PMC5660876; doi:10.7717/peerj.3941)
Supplement: Table S1 — Diversity of the Chilean clade of Liolaemus (species and groups) adapted and updated from Abdala & Quinteros (2013). Synonyms and additions relative to the classification proposed by Abdala & Quinteros (2013) are shown. The recently described Liolaemus scorialis Troncoso-Palacios et al. (2015) is incertae sedis in the elongatus-kriegi complex (Troncoso-Palacios et al., 2015). Species included in this study are marked in bold. [file peerj-05-3941-s001.docx]

|  | ***sensu* Abdala & Quinteros, 2013** | **Synonym** | **Additions** |
| --- | --- | --- | --- |
| *alticolor–bibronii* group | *L. alticolor* |  |  |
|  | *L. abdalai* |  |  |
|  | *L. aparicioi* |  |  |
|  | *L. araucaniensis* |  |  |
|  | *L. bibronii* |  |  |
|  | *L. bitaeniatus* |  |  |
|  | *L. chaltin* |  |  |
|  | *L. curicensis* |  |  |
|  | *L. cyaneinotatus* |  |  |
|  | *L. exploratorum* |  |  |
|  | ***L. fuscus*** |  |  |
|  | *L. gracilis* |  |  |
|  | *L. incaicus* |  |  |
|  | *L. lativitattus* | Synonimized with *L. alticolor* sensu Müller & Hellmich (1938), not accepted by Lobo, Espinoza & Quinteros (2010) and Quinteros (2012). |  |
|  | *L. lemniscatus* |  |  |
|  | *L. pagaburoi* |  |  |
|  | ***L. paulinae*** |  |  |
|  | *L. puna* |  |  |
|  | *L. pyriphlogos* |  |  |
|  | *L. ramirezae* |  |  |
|  | *L. saxatilis* |  |  |
|  | *L. tacnae* |  |  |
|  | *L. tandiliensis* |  |  |
|  | *L. variegatus* |  |  |
|  | *L. walkeri* |  |  |
|  | *L. yanalcu* |  |  |
|  |  |  | *L. yalguaraz* Abdala et al., 2015 |
| *bellii* group | *L. bellii* |  |  |
|  | *L. curis* |  |  |
|  | *L. fitzgeraldi* |  |  |
|  | *L. modestus* | Is now *Stenocercus modestus* (Tschudi, 1845) (Laurent, 1984; Torres-Carvajal, 2007). |  |
|  | *L. moradoensis* |  |  |
| *capillitas* group | *L. capillitas* |  |  |
|  | *L. dicktracyi* |  |  |
|  | *L. heliodermis* |  |  |
|  | *L. talampaya* |  |  |
|  | *L. tulkas* |  |  |
|  | *L. umbrifer* |  |  |
| *chillanensis* group | *L. chillanensis* |  |  |
|  | *L. villaricensis* |  |  |
|  |  |  | *L. leftrarui* Troncoso-Palacios et al., (2016a) |
|  |  |  |  |
|  |  |  |  |
| *elongatus* group | *L. austromendocinus* |  |  |
|  | *L. antumalguen* |  |  |
|  | *L. burmeisteri* |  |  |
|  | *L. carlosgarini* |  |  |
|  | *L. choique* |  |  |
|  | *L. elongatus* |  |  |
|  | *L. flavipiceus* |  |  |
|  | *L. gununakuna* |  |  |
|  | *L. parvus* |  |  |
|  | *L. petrophilus* |  |  |
|  | *L. punmahuida* |  |  |
|  | *L. riodamas* |  |  |
|  | *L. shitan* |  |  |
|  | *L. smaug* |  |  |
|  | *L. thermarum* |  |  |
|  | *L. tregenzai* |  |  |
|  |  |  | *L. janequeoae* Troncoso-Palacios et al., (2016a) |
| *gravenhorstii* group | ***L. cyanogaster*** |  |  |
|  | *L. gravenhorsti* |  |  |
|  | *L. schroederi* |  |  |
| *kriegi* group | *L. buergeri* |  |  |
|  | *L. ceii* | Synonymized with *L. kriegi* by Morando et al., (2003) and Troncoso-Palacios et al., (2015) |  |
|  | *L. cristiani* |  |  |
|  | *L. kriegi* |  |  |
|  |  |  | *L. zabalai* Troncoso-Palacios et al., (2015) |
| *leopardinus* group | *L. frassinettii* |  |  |
|  | *L. leopardinus* |  |  |
|  | *L. ramonensis* |  |  |
|  | *L. valdesianus* |  |  |
|  |  |  | *L. ubaghsi* Esquerré et al., 2014 |
| *monticola* group | ***L. monticola*** |  |  |
|  | *L. confusus* |  |  |
| *nigromaculatus* group | ***L. atacamensis*** |  |  |
|  | *L. ater* | *L. ater* is currently considered subspecies of *L. zapallarensis* (Ortiz, 1981). |  |
|  | *L. bisignatus* | *Liolaemus bisignatus is* a *nomen nudum*, and populations attributed to *L. bisignatus* should be referred to *L. nigromaculatus* (Troncoso-Palacios & Garín, 2013). |  |
|  | *L. copiapensis* | *L. copiapensis* is a synonym of *L.bisignatus* (Pincheira-Donoso & Núñez, 2005; Troncoso-Palacios & Garín, 2013), which is in turn synonym to *L. nigromaculatus* (Troncoso-Palacios & Garín, 2013). |  |
|  | *L. donosoi* | *L. donosoi* is currently considered synonym of *L. constanzae* (Veloso et al., 1982; Troncoso-Palacios, 2013). |  |
|  | *L. hellmichi* |  |  |
|  | *L. kuhlmanni* | Pincheira-Donoso et al., (2008) considered *L. kuhlmanni* as a synonym of *L. zapallarensis*, a decision that is rejected by Lobo, Espinoza & Quinteros (2010) and Ruiz de Gamboa (2016). |  |
|  | *L. melaniceps* |  |  |
|  | ***L. nigromaculatus*** |  |  |
|  | ***L. platei*** |  |  |
|  | *L. pseudolemniscatus* |  |  |
|  | *L. sieversi* | *L. sieversi* is currently considered subspecies of *L. zapallarensis* (Ortiz, 1981). |  |
|  | *L. silvai* |  |  |
|  | ***L. velosoi*** |  |  |
|  | ***L. zapallarensis*** |  |  |
|  |  |  | *L. nigrocoeruleus* Marambio-Alfaro & Troncoso-Palacios (2014) |
| *nigroviridis* group | *L. constanzae* |  |  |
|  | ***L. isabelae*** |  |  |
|  | *L. juanortizi* |  |  |
|  | *L. lorenzmuelleri* |  |  |
|  | *L. maldonadae* |  |  |
|  | *L. melanopleurus* |  |  |
|  | ***L. nigroviridis*** |  |  |
|  |  |  | *L. uniformis* Troncoso-Palacios et al., (2016b) |
| *pictus* group | *L. brattstroemi* | Pincheira-Donoso & Núñez (2005) elevate *L. cyanogaster brattstroemi* to full species. Lobo, Espinoza & Quinteros (2010) support this decision while Vidal et al., (2012) & Ruiz de Gamboa (2016) disagree. |  |
|  | *L. argentinus* | Is a subspecies of *L. pictus* (Ruiz de Gamboa, 2016) |  |
|  | *L. chiloensis* | It was formerly a subspecies of *L. pictus* although Vera-Escalona et al., (2012) synonymized it to *L. pictus.* |  |
|  | *L. codoceae* | Is a subspecies of *L. pictus* (Pincheira-Donoso & Núñez, 2005) |  |
|  | *L. major* | Synonimized with *L. pictus codoceae* by Pincheira-Donoso & Núñez (2005) and supported by Lobo, Espinoza & Quinteros (2010) |  |
|  | ***L. pictus*** |  |  |
|  | *L. septentrionalis* | Pincheira-Donoso et al., (2008) elevated *L. pictus septentrionalis* to full species, which has been supported by Lobo, Espinoza & Quinteros (2010) and Vera-Escalona et al., (2012). |  |
|  | *L. talcanensis* | Is a subspecies of *L. pictus* (Pincheira-Donoso & Núñez, 2005) |  |
|  | ***L. tenuis*** |  |  |
|  | *L. coeruleus* |  |  |
|  | *L. neuquensis* |  |  |
| *robertmertensi* group | *L. chiliensis* |  |  |
|  | ***L. nitidus*** |  |  |
|  | *L. robertmertensi* |  |  |
|  | *L. sanjuanensis* |  |  |

REFERENCES

Abdala, C. S. & Quinteros, A. S. (2013). Los últimos 30 años de estudios de la familia de lagartijas más diversa de Argentina. Actualización taxonómica y sistemática de Liolaemidae. *Cuadernos de Herpetología*, 28, 1-28.

Abdala, C. S., Quinteros, A. S. & Semham, R. V. (2015). A new species of *Liolaemus* of the *Liolaemus alticolor-bibronii* group (Iguania: Liolaemidae) from Mendoza, Argentina. *South American Journal of Herpetology*, 10(2), 104-115.

Esquerre, D., Troncoso-Palacios, J., Garin, C. F. & Nunez, H. (2014). The missing leopard lizard: *Liolaemus ubaghsi* sp. nov., a new species of the *leopardinus* clade (Reptilia: Squamata: Liolaemidae) from the Andes of the O’Higgins Region in Chile. *Zootaxa*, 3815(4), 507-525.

Laurent, R. F. (1984). On some iguanid genera related to or previously confused with *Liolaemus* Wiegmann. *Journal of Herpetology*, 357-373.

Lobo, F., Espinoza, R. E. & Quinteros, S. (2010). A critical review and systematic discussion of recent classification proposals for liolaemid lizards. *Zootaxa*, 2549, 1-30.

Marambio-Alfaro, Y. & Troncoso-Palacios, J. (2014). A new species of *Liolaemus* of the *L. nigromaculatus* group (Iguania: Liolaemidae) from Atacama Region, Chile. *Basic and Applied Herpetology*, 28, 65-77.

Morando, M., Avila, L. J. & Sites, J. W. (2003). Sampling strategies for delimiting species: genes, individuals, and populations in the *Liolaemus elongatus-kriegi* complex (Squamata: Liolaemidae) in Andean–Patagonian South America. *Systematic Biology*, 52(2), 159-185.

Müller, L., & Hellmich, W. (1938). Liolaemus-Arten aus dem westlichen Argentinien (ergebnisse der argentinienreise von Prof. Dr. W. Goetsch, Breslau). I. *Liolaemus darwini* (Bell) und *Liolaemus goetschi* n. sp. *Zoologischer Anzeiger*, 123(5-6), 130-142.

Ortiz, J.C. (1981). Estudio multivariado de las especies de *Liolaemus* del grupo *nigromaculatus* (Squamata, Iguanidae). *Anales del Museo de Historia Natural de Valparaíso*, 14, 247-265.

Pincheira-Donoso, D. & Núñez, H. (2005). Las especies chilenas del género *Liolaemus* Wiegmann, 1834 (Iguania Tropiduridae: Liolaeminae). Taxonomía, sistemática y evolución. *Publicación ocasional del Museo Nacional de Historia Natural,* 59, 7-486.

Pincheira-Donoso, D., Scolaro, J. A. & Sura, P. (2008). A monographic catalogue on the systematics and phylogeny of the South American iguanian lizard family Liolaemidae (Squamata, Iguania). *Zootaxa*, 1800, 1–85.

Quinteros, A. S. (2012). Taxonomy of the *Liolaemus alticolor-bibronii* group (Iguania: Liolaemidae), with descriptions of two new species. *Herpetologica*, 68(1), 100-120.

Ruiz de Gamboa, M. (2016). Lista actualizada de los reptiles de Chile. *Boletín Chileno de Herpetología*, 3, 7-12.

Torres-Carvajal, O. (2007). A taxonomic revision of South American *Stenocercus* (Squamata: iguania) lizards. *Herpetological monographs*, 21(1), 76-178.

Troncoso-Palacios, J. (2013). Revisión del estatus taxonómico *de Liolaemus donosoi* Ortiz, 1975 (Iguania: Liolaemidae). *Boletín del Museo Nacional de Historia Natural de Chile*, 62, 119-127.

Troncoso-Palacios, J. & Garin, C. F. (2013). On the identity of *Liolaemus nigromaculatus* Wiegmann, 1834 (Iguania, Liolaemidae) and correction of its type locality. *ZooKeys*, 294, 37.

Troncoso-Palacios, J., Díaz, H. A., Esquerré, D. & Urra, F. A. (2015). Two new species of the *Liolaemus elongatus-kriegi* complex (Iguania, Liolaemidae) from Andean highlands of southern Chile. *ZooKeys*, 500, 83.

Troncoso-Palacios, J., Diaz, H. A., Puas, G. I., Riveros-Riffo, E. & Elorza, A. A. (2016a). Two new *Liolaemus* lizards from the Andean highlands of Southern Chile (Squamata, Iguania, Liolaemidae). *ZooKeys*, 632, 121.

Troncoso-Palacios, J., Elorza, A. A., Puas, G. I. & Alfaro-Pardo, E. (2016b). A new species of *Liolaemus* related to *L. nigroviridis* from the Andean highlands of Central Chile (Iguania, Liolaemidae). *ZooKeys*, (555), 91.

Veloso, A., Sallaberry, M., Navarro, J., Iturra, P., Valencia, J., Penna, M. & Díaz, Y.N. (1982) Contribución sistemática al conocimiento de la herpetofauna del extremo norte de Chile. In Veloso, A. y E. Bustos (Eds.). El hombre y los ecosistemas de montaña. Oficina Regional de Ciencias y Tecnología de la UNESCO para América Latina y el Caribe. (Montevideo, Uruguay). I, 135-268

Vera-Escalona, I., D'Elía, G., Gouin, N., Fontanella, F. M., Muñoz-Mendoza, C., Sites Jr, J. W. & Victoriano, P. F. (2012). Lizards on ice: evidence for multiple refugia in *Liolaemus pictus* (Liolaemidae) during the last glacial maximum in the southern Andean beech forests. *PloS one*, 7(11), e48358.

Vidal, M. A., Ortiz, J. C., Marín, J. C., Poulin, E. & Moreno, P. I. (2012). Comparative phylogeography of two co-distributed species of lizards of the genus *Liolaemus* (Squamata: Tropiduridae) from Southern Chile. *Amphibia-Reptilia*, 33(1), 55-67.
