# Supplementary material for: Phylogenomic analysis of the Chilean clade of Liolaemus lizards (Squamata: Liolaemidae) based on sequence capture data
Source: PeerJ. 2017 Oct 26;5:e3941. doi: 10.7717/peerj.3941 (PMC5660876; doi:10.7717/peerj.3941)
Supplement: Table S2 — Detailed locality data for the Chilean Liolaemus species included in the study. Coordinates are given in decimal degrees. Detailed type locality information, including coordinates when available, as well as bibliographic references are given. [file peerj-05-3941-s002.docx]

| **Species** | **Voucher** | **Locality** | **Latitude** | **Longitude** | **Type locality** | **Source** |
| --- | --- | --- | --- | --- | --- | --- |
| *L. atacamensis* | MZUC-45084 | Entre Finca de Chañaral e Inca de Oro, Km 10, ruta c-253 | -26,6784 | -69,8441 | Atacama, north of Copiapó | Müller & Hellmich, 1933 |
| *L. cyanogaster* | MZUC-45092 | Estación San Pedro | -39,7582 | -72,6728 | Chile, and restricted to Valdivia afterwards | Hellmich, 1934 |
| *L. fuscus* | MZUC-45085 | La Herradura, Coquimbo | -29,9792 | -71,3713 | Valparaíso | Boulenger, 1885 |
| *L. isabelae* | MZUC-45086 | Ruta c-189, pasando Salar de Pedernales hacia Diego de Almagro | -26,4001 | -69,2808 | El Cerrito, 12 km North-West from campamento La Ola, near Salar de Pedernales (26°27' S, 69°03 W/26°12' S, 69°08 W) | Navarro & Núñez, 1993 |
| *L. monticola* | LMON619 | El Yeso | -33,7232 | -70,6169 | San Francisco river, 1700 m. altitude (32°22′S, 70°25′W) | Torres-Pérez et al., 2009 |
| *L. nigromaculatus* | SSUC 643 | Las Terrazas Beach, Paposo | -25,14 | -70,4613 | Between Puerto Viejo and Copiapó | Troncoso-Palacios & Garín, 2013 |
| *L. nigroviridis* | LNIG614 | El Yeso | -33,7232 | -70,6169 | San Francisco river (32°22' S, 70°25' W) | Cianferoni et al., 2013 |
| *L. nitidus* | MZUC-45087 | 2 km north of Algarrobo | -33,3601 | -71,6463 | Chile, and restricted to Valparaíso afterwards | Hellmich, 1934 |
| *L. paulinae* | MZUC-45088 | Rio Loa, Calama | -22.4784 | -68.9156 | Calama on the Loa River, 2600 m. altitude. | Donoso-Barros, 1961 |
| *L. pictus* | MZUC-45094 | Valdivia National Park (Chaiguin) | -39,9504 | -73,5601 | Chile, and restricted to Valdivia afterwards | Hellmich, 1934 |
| *L. platei* | MZUC-45089 | Quebrada Buenos Aires | -29,57036 | -71,24391 | Coquimbo | Werner, 1898 |
| *L. sp.* | BYU 49951 | Caracol | -36,6567 | -71,3656 | - | - |
| *L. t. punctatissimus* | BYU 48375 | Playa Negra, Lota-Coronel | -37,0833 | -73,1667 | Lota (37°04 S, 73°10 W) | Donoso-Barros, 1966 |
| *L. t. tenuis* | MZUC-45093 | Tiltil | -33,1355 | -70,8552 | Chile, and restricted to Santiago afterwards | Hellmich, 1934 |
| *L. velosoi* | MZUC-45090 | Nantoco | -27,57215 | -70,25961 | Desvio Cerro Imán près de Copiapó (27°20’ S 70°30 W) | Ortiz, 1987 |
| *L. zapallarensis* | MZUC-45091 | Quebrada Buenos Aires | -29,57036 | -71,24391 | Zapallar | Müller & Hellmich, 1933 |

REFERENCES

Boulenger, G.A. (1885). *Catalogue of the lizards in the British Museum (Natural History*). Second edition. London.

Cianferoni, F., Yanez, R. P., Palma, R. E., Garin, C. F. & Torres-Perez, F. (2013). Deep divergences within *Liolaemus nigroviridis* (Squamata, Liolaemidae) lineages associated with sky islands in central Chile. *Zootaxa*, 3619, 59-69

Donoso-Barros, R. (1961). Three new lizards of the genus *Liolaemus* from the highest Andes of Chile and Argentina. *Copeia*, 1961(4), 387-391.

Donoso-Barros, R. (1966). *Reptiles of Chile*. Santiago: Editorial Universitaria.

Hellmich, W. (1934). Die Eidechsen Chiles insbesonders die Gattung *Liolaemus*. Nach den Sammlungen Goetsch-Hellmich. Abhandlungen. Bayerischen Akademie der issenschaften. *Mathematisch-Naturwissenschaftliche Klasse,* 24, 1–140

Müller, L. & Hellmich, W. (1933). Beiträge zur Kenntnis der Herpetofauna Chiles. VII. Der Rassenkreis *der Liolaemus nigromaculatus*. *Zoologischer Anzeiger*, 103, 128-142.

Navarro, J. & Núñez, H. (1993). *Liolaemus patriciaiturrae* y *Liolaemus isabelae*, dos nuevas especies de lagartijas para el norte de Chile. Aspectos biogeográficos y citotaxonómicos (Squamata, Tropiduridae). *Boletín del Museo Nacional de Historia Natural de Chile*, 44, 99-113.

Ortiz, J.C. (1987). Une nouvelle espèce de *Liolaemus* (Sauria, Iguanidae) du Chili. *Bulletin Museum National d'Histoire Naturelle*, *Paris*, 265-270.

Torres-Pérez F., Méndez M.A., Benavides E., Moreno R.A., Lamborot M., Palma R.E. & J.C. Ortiz. (2009). Systematics and evolutionary relationships of the mountain lizard *Liolaemus monticola* (Liolaemini): how morphological and molecular evidence contributes to reveal hidden species diversity. *Biological Journal of the Linnean Society,* 96, 635–650.

Troncoso-Palacios, J. & Garin, C. F. (2013). On the identity of *Liolaemus nigromaculatus* Wiegmann, 1834 (Iguania, Liolaemidae) and correction of its type locality. *ZooKeys*, 294, 37.

Werner, F. (1898). Die Reptilien und Batrachier der Sammlung Plate. *Zoologische Jahrbuecher Abteilung fuer Systematik Oekologie und Geographie der Tiere*, 4, 244-278.
