# Supplementary material for: Phylogenomic analysis of the Chilean clade of Liolaemus lizards (Squamata: Liolaemidae) based on sequence capture data
Source: PeerJ. 2017 Oct 26;5:e3941. doi: 10.7717/peerj.3941 (PMC5660876; doi:10.7717/peerj.3941)
Supplement: Table S3 — Comparison of de novo assembly results from IDBA across different k-mer values. The k-mer value with the highest N50 was chosen for each species (shown in bold). One exception is L. t. tenuis, which had too few loci. [file peerj-05-3941-s003.docx]

|  | K-mer value | | | | | | | | | |
| --- | --- | --- | --- | --- | --- | --- | --- | --- | --- | --- |
|  | **50** | | **60** | | **70** | | **80** | | **90** | |
| Species | **Contigs** | **N50** | **Contigs** | **N50** | **Contigs** | **N50** | **Contigs** | **N50** | **Contigs** | **N50** |
| *L. atacamensis* | 8,020 | 158 | 2,857 | 213 | 1,651 | 283 | 1,085 | 358 | **949** | **388** |
| *L. cyanogaster* | 12,715 | 150 | 4,162 | 216 | 2,464 | 294 | **1,780** | **357** | 1,813 | 346 |
| *L. fuscus* | 6,055 | 131 | 3,181 | 160 | 1,846 | 225 | 1,240 | 293 | **1,044** | **326** |
| *L. isabelae* | 20,852 | 102 | 9,477 | 121 | 4,306 | 178 | **2,150** | **257** | 2,707 | 221 |
| *L. monticola* | 26,585 | 119 | 10,697 | 145 | 5,034 | 205 | 2,906 | 263 | **2,803** | **331** |
| *L. nigromaculatus* | 2,640 | 148 | 1,115 | 187 | 625 | 249 | 478 | 281 | **428** | **286** |
| *L. nigroviridis* | 1,746,196 | 58 | 1,246,148 | 69 | 741,647 | 83 | 399,483 | 97 | 253,864 | 111 |
| *L. nitidus* | 1,169,208 | 62 | 862,813 | 72 | 522,466 | 86 | 276,282 | 103 | **272,920** | **112** |
| *L. paulinae* | 333,832 | 204 | 192,398 | 255 | 141,917 | 292 | **111,191** | **320** | 101,712 | 317 |
| *L. pictus* | 6,463 | 140 | 1,408 | 217 | 898 | 273 | 641 | 322 | **574** | **331** |
| *L. platei* | 16,473 | 131 | 7,379 | 151 | 3,650 | 205 | **2,160** | **257** | 2,026 | 253 |
| *L. t. punctatissimus* | 11,397 | 97 | 3,523 | 130 | 1,238 | 204 | 661 | 277 | **517** | **307** |
| *L. sp.* | 605 | 189 | 250 | 279 | 202 | 304 | 171 | 319 | **144** | **327** |
| *L. t. tenuis* | **235** | **133** | 62 | 157 | 33 | 167 | 11 | 213 | 3 | 232 |
| *L. velosoi* | 271,652 | 160 | 131,141 | 204 | 82,958 | 252 | **57,514** | **293** | 51,813 | 290 |
| *L. zapallarensis* | 167,794 | 120 | 92,148 | 138 | 45,614 | 192 | **27,903** | **239** | 26,430 | 235 |
