# Supplementary material for: Phylogenomic analysis of the Chilean clade of Liolaemus lizards (Squamata: Liolaemidae) based on sequence capture data
Source: PeerJ. 2017 Oct 26;5:e3941. doi: 10.7717/peerj.3941 (PMC5660876; doi:10.7717/peerj.3941)
Supplement: Table S4 [file peerj-05-3941-s004.docx]

| Type | Marker | Inf. Sites | % Inf. Sites |
| --- | --- | --- | --- |
| UCE | chr12_1169 | 0 | 0.0% |
| UCE | chr12_1475 | 5 | 1.1% |
| UCE | chr12_2213 | 8 | 1.8% |
| UCE | chr12_2426 | 2 | 0.5% |
| UCE | chr12_3124 | 0 | 0.0% |
| UCE | chr12_3154 | 3 | 1.0% |
| UCE | chr12_3865 | 0 | 0.0% |
| UCE | chr12_5665 | 2 | 0.5% |
| UCE | chr12_5671 | 1 | 0.3% |
| UCE | chr12_5730 | 0 | 0.0% |
| UCE | chr12_5739 | 0 | 0.0% |
| UCE | chr12_5828 | 0 | 0.0% |
| UCE | chr12_5837 | 4 | 0.7% |
| UCE | chr12_5840 | 2 | 0.5% |
| UCE | chr12_5851 | 2 | 0.5% |
| UCE | chr12_5878 | 8 | 2.1% |
| UCE | chr12_5895 | 3 | 0.7% |
| UCE | chr12_5903 | 0 | 0.0% |
| UCE | chr12_5908 | 0 | 0.0% |
| UCE | chr12_5912 | 12 | 2.5% |
| UCE | chr12_5949 | 6 | 2.0% |
| UCE | chr12_5969 | 6 | 1.6% |
| UCE | chr13_1225 | 0 | 0.0% |
| UCE | chr13_4268 | 9 | 4.1% |
| UCE | chr13_5059 | 2 | 0.6% |
| UCE | chr13_5324 | 2 | 0.6% |
| UCE | chr13_710 | 2 | 0.4% |
| UCE | chr13_720 | 0 | 0.0% |
| UCE | chr13_726 | 0 | 0.0% |
| UCE | chr18_1422 | 3 | 0.8% |
| UCE | chr18_4493 | 0 | 0.0% |
| UCE | chr1_10949 | 2 | 0.5% |
| UCE | chr1_13047 | 3 | 0.8% |
| UCE | chr1_13198 | 4 | 1.0% |
| UCE | chr1_13698 | 2 | 0.5% |
| UCE | chr1_1378 | 4 | 1.1% |
| UCE | chr1_1418 | 0 | 0.0% |
| UCE | chr1_14389 | 3 | 0.7% |
| UCE | chr1_14672 | 6 | 1.5% |
| UCE | chr1_15412 | 2 | 0.6% |
| UCE | chr1_15480 | 3 | 0.8% |
| UCE | chr1_15632 | 4 | 1.1% |
| UCE | chr1_16199 | 3 | 0.8% |
| UCE | chr1_18915 | 3 | 0.7% |
| UCE | chr1_19202 | 3 | 0.8% |
| UCE | chr1_19246 | 2 | 0.5% |
| UCE | chr1_19292 | 1 | 0.3% |
| UCE | chr1_19426 | 1 | 0.2% |
| UCE | chr1_19885 | 2 | 0.5% |
| UCE | chr1_21570 | 0 | 0.0% |
| UCE | chr1_22432 | 3 | 0.7% |
| UCE | chr1_23573 | 10 | 2.6% |
| UCE | chr1_24625 | 0 | 0.0% |
| UCE | chr1_24640 | 2 | 0.4% |
| UCE | chr1_24644 | 2 | 0.6% |
| UCE | chr1_24817 | 0 | 0.0% |
| UCE | chr1_25630 | 2 | 0.6% |
| UCE | chr1_25670 | 0 | 0.0% |
| UCE | chr1_25675 | 0 | 0.0% |
| UCE | chr1_25680 | 0 | 0.0% |
| UCE | chr1_25692 | 1 | 0.3% |
| UCE | chr1_25699 | 1 | 0.3% |
| UCE | chr1_25705 | 4 | 0.9% |
| UCE | chr1_26035 | 0 | 0.0% |
| UCE | chr1_27509 | 7 | 2.0% |
| UCE | chr1_27552 | 2 | 0.4% |
| UCE | chr1_2930 | 7 | 2.2% |
| UCE | chr1_29790 | 0 | 0.0% |
| UCE | chr1_29835 | 0 | 0.0% |
| UCE | chr1_29841 | 1 | 0.2% |
| UCE | chr1_29894 | 10 | 2.2% |
| UCE | chr1_29912 | 1 | 0.3% |
| UCE | chr1_30195 | 3 | 0.8% |
| UCE | chr1_30635 | 0 | 0.0% |
| UCE | chr1_31673 | 2 | 0.5% |
| UCE | chr1_31677 | 2 | 0.5% |
| UCE | chr1_31709 | 1 | 0.3% |
| UCE | chr1_31743 | 2 | 0.5% |
| UCE | chr1_31749 | 3 | 0.8% |
| UCE | chr1_31783 | 7 | 1.7% |
| UCE | chr1_32194 | 7 | 1.8% |
| UCE | chr1_32208 | 4 | 1.0% |
| UCE | chr1_32232 | 5 | 1.2% |
| UCE | chr1_32234 | 4 | 1.0% |
| UCE | chr1_32266 | 11 | 2.9% |
| UCE | chr1_32286 | 2 | 0.6% |
| UCE | chr1_32322 | 1 | 0.2% |
| UCE | chr1_32333 | 7 | 1.6% |
| UCE | chr1_32337 | 1 | 0.3% |
| UCE | chr1_32356 | 3 | 0.8% |
| UCE | chr1_32365 | 0 | 0.0% |
| UCE | chr1_32370 | 1 | 0.3% |
| UCE | chr1_32378 | 5 | 1.4% |
| UCE | chr1_32429 | 1 | 0.2% |
| UCE | chr1_32443 | 0 | 0.0% |
| UCE | chr1_32461 | 4 | 1.1% |
| UCE | chr1_33834 | 6 | 1.8% |
| UCE | chr1_34776 | 5 | 1.3% |
| UCE | chr1_3857 | 3 | 0.8% |
| UCE | chr1_4680 | 3 | 0.8% |
| UCE | chr1_5277 | 1 | 0.2% |
| UCE | chr1_5279 | 1 | 0.3% |
| UCE | chr1_5288 | 0 | 0.0% |
| UCE | chr1_5301 | 4 | 1.1% |
| UCE | chr1_5319 | 0 | 0.0% |
| UCE | chr1_5334 | 0 | 0.0% |
| UCE | chr1_5365 | 2 | 0.5% |
| UCE | chr1_5379 | 0 | 0.0% |
| UCE | chr1_5409 | 0 | 0.0% |
| UCE | chr1_5426 | 1 | 0.2% |
| UCE | chr1_5466 | 1 | 0.3% |
| UCE | chr1_5470 | 0 | 0.0% |
| UCE | chr1_5474 | 1 | 0.2% |
| UCE | chr1_5479 | 0 | 0.0% |
| UCE | chr1_5492 | 0 | 0.0% |
| UCE | chr1_8658 | 2 | 0.5% |
| UCE | chr1_8991 | 3 | 0.8% |
| UCE | chr20_1391 | 1 | 0.2% |
| UCE | chr20_253 | 2 | 0.5% |
| UCE | chr20_3629 | 8 | 2.2% |
| UCE | chr26_2189 | 11 | 2.5% |
| UCE | chr26_2766 | 1 | 0.3% |
| UCE | chr26_2850 | 0 | 0.0% |
| UCE | chr2_11187 | 6 | 2.0% |
| UCE | chr2_11494 | 3 | 0.9% |
| UCE | chr2_11510 | 3 | 1.0% |
| UCE | chr2_11732 | 2 | 0.5% |
| UCE | chr2_11743 | 7 | 1.7% |
| UCE | chr2_11785 | 2 | 0.5% |
| UCE | chr2_11789 | 3 | 0.7% |
| UCE | chr2_11804 | 1 | 0.2% |
| UCE | chr2_12928 | 3 | 0.9% |
| UCE | chr2_12992 | 2 | 0.5% |
| UCE | chr2_12994 | 3 | 0.7% |
| UCE | chr2_13030 | 2 | 0.5% |
| UCE | chr2_13032 | 2 | 0.5% |
| UCE | chr2_13034 | 1 | 0.3% |
| UCE | chr2_13064 | 1 | 0.2% |
| UCE | chr2_13460 | 6 | 1.8% |
| UCE | chr2_13502 | 6 | 1.4% |
| UCE | chr2_1647 | 0 | 0.0% |
| UCE | chr2_17005 | 3 | 0.7% |
| UCE | chr2_17019 | 3 | 0.9% |
| UCE | chr2_17532 | 1 | 0.2% |
| UCE | chr2_18468 | 7 | 1.8% |
| UCE | chr2_18477 | 2 | 0.5% |
| UCE | chr2_18557 | 4 | 0.9% |
| UCE | chr2_18578 | 0 | 0.0% |
| UCE | chr2_18589 | 2 | 0.5% |
| UCE | chr2_18608 | 0 | 0.0% |
| UCE | chr2_18614 | 12 | 2.9% |
| UCE | chr2_18619 | 1 | 0.3% |
| UCE | chr2_18662 | 5 | 1.4% |
| UCE | chr2_18677 | 0 | 0.0% |
| UCE | chr2_18686 | 2 | 0.5% |
| UCE | chr2_18714 | 2 | 0.6% |
| UCE | chr2_18743 | 0 | 0.0% |
| UCE | chr2_1916 | 1 | 0.2% |
| UCE | chr2_20477 | 2 | 0.5% |
| UCE | chr2_21229 | 1 | 0.3% |
| UCE | chr2_21265 | 0 | 0.0% |
| UCE | chr2_21284 | 2 | 0.6% |
| UCE | chr2_21308 | 1 | 0.2% |
| UCE | chr2_21320 | 2 | 0.5% |
| UCE | chr2_21344 | 6 | 1.6% |
| UCE | chr2_21358 | 4 | 1.2% |
| UCE | chr2_21401 | 1 | 0.3% |
| UCE | chr2_21445 | 1 | 0.3% |
| UCE | chr2_2239 | 9 | 2.7% |
| UCE | chr2_23113 | 5 | 1.2% |
| UCE | chr2_23160 | 2 | 0.5% |
| UCE | chr2_23221 | 4 | 1.2% |
| UCE | chr2_23596 | 1 | 0.2% |
| UCE | chr2_23621 | 2 | 0.5% |
| UCE | chr2_23635 | 1 | 0.2% |
| UCE | chr2_23648 | 13 | 3.5% |
| UCE | chr2_23668 | 0 | 0.0% |
| UCE | chr2_24173 | 1 | 0.3% |
| UCE | chr2_24655 | 4 | 0.9% |
| UCE | chr2_24672 | 9 | 2.0% |
| UCE | chr2_24684 | 3 | 0.7% |
| UCE | chr2_24697 | 5 | 1.3% |
| UCE | chr2_24704 | 3 | 0.9% |
| UCE | chr2_24800 | 0 | 0.0% |
| UCE | chr2_24815 | 0 | 0.0% |
| UCE | chr2_24827 | 3 | 0.8% |
| UCE | chr2_24841 | 1 | 0.2% |
| UCE | chr2_24859 | 5 | 1.2% |
| UCE | chr2_24876 | 3 | 0.8% |
| UCE | chr2_24879 | 2 | 0.5% |
| UCE | chr2_24910 | 2 | 0.6% |
| UCE | chr2_25833 | 6 | 1.6% |
| UCE | chr2_25851 | 5 | 1.5% |
| UCE | chr2_27241 | 3 | 0.7% |
| UCE | chr2_27258 | 3 | 0.7% |
| UCE | chr2_27261 | 2 | 0.5% |
| UCE | chr2_27280 | 1 | 0.3% |
| UCE | chr2_27294 | 0 | 0.0% |
| UCE | chr2_27313 | 0 | 0.0% |
| UCE | chr2_27968 | 3 | 0.8% |
| UCE | chr2_29406 | 1 | 0.3% |
| UCE | chr2_4395 | 0 | 0.0% |
| UCE | chr2_5491 | 1 | 0.3% |
| UCE | chr2_5499 | 0 | 0.0% |
| UCE | chr2_5526 | 2 | 0.6% |
| UCE | chr2_5991 | 1 | 0.2% |
| UCE | chr2_6341 | 1 | 0.3% |
| UCE | chr2_6444 | 0 | 0.0% |
| UCE | chr2_6685 | 0 | 0.0% |
| UCE | chr2_6737 | 2 | 0.6% |
| UCE | chr2_6787 | 0 | 0.0% |
| UCE | chr2_7409 | 0 | 0.0% |
| UCE | chr2_7420 | 3 | 0.6% |
| UCE | chr2_7927 | 0 | 0.0% |
| UCE | chr2_7945 | 3 | 0.7% |
| UCE | chr2_7954 | 1 | 0.3% |
| UCE | chr2_8583 | 2 | 0.5% |
| UCE | chr2_8589 | 3 | 0.7% |
| UCE | chr2_8590 | 0 | 0.0% |
| UCE | chr2_8600 | 2 | 0.6% |
| UCE | chr2_8609 | 1 | 0.3% |
| UCE | chr2_8620 | 2 | 0.6% |
| UCE | chr2_8629 | 5 | 1.2% |
| UCE | chr2_8651 | 9 | 2.6% |
| UCE | chr2_8655 | 5 | 1.0% |
| UCE | chr2_8677 | 3 | 0.8% |
| UCE | chr2_8688 | 1 | 0.3% |
| UCE | chr2_8698 | 3 | 0.8% |
| UCE | chr2_8747 | 1 | 0.2% |
| UCE | chr2_8754 | 1 | 0.3% |
| UCE | chr3_11795 | 1 | 0.3% |
| UCE | chr3_11879 | 9 | 2.3% |
| UCE | chr3_1282 | 3 | 0.8% |
| UCE | chr3_1300 | 1 | 0.2% |
| UCE | chr3_13359 | 5 | 1.4% |
| UCE | chr3_13404 | 1 | 0.3% |
| UCE | chr3_16820 | 3 | 0.8% |
| UCE | chr3_16833 | 4 | 1.1% |
| UCE | chr3_17448 | 10 | 2.3% |
| UCE | chr3_17607 | 1 | 0.3% |
| UCE | chr3_17623 | 3 | 0.7% |
| UCE | chr3_17699 | 2 | 0.5% |
| UCE | chr3_17721 | 0 | 0.0% |
| UCE | chr3_17747 | 3 | 0.7% |
| UCE | chr3_17769 | 4 | 1.0% |
| UCE | chr3_17781 | 0 | 0.0% |
| UCE | chr3_17860 | 2 | 0.6% |
| UCE | chr3_17890 | 2 | 0.5% |
| UCE | chr3_17991 | 5 | 1.3% |
| UCE | chr3_18203 | 1 | 0.2% |
| UCE | chr3_18256 | 1 | 0.3% |
| UCE | chr3_18306 | 1 | 0.2% |
| UCE | chr3_19568 | 0 | 0.0% |
| UCE | chr3_19997 | 2 | 0.5% |
| UCE | chr3_20013 | 6 | 1.9% |
| UCE | chr3_21510 | 14 | 3.3% |
| UCE | chr3_21949 | 4 | 1.1% |
| UCE | chr3_22024 | 1 | 0.3% |
| UCE | chr3_23724 | 1 | 0.3% |
| UCE | chr3_24903 | 3 | 0.8% |
| UCE | chr3_24963 | 5 | 1.3% |
| UCE | chr3_25095 | 7 | 2.0% |
| UCE | chr3_2698 | 5 | 1.3% |
| UCE | chr3_2723 | 0 | 0.0% |
| UCE | chr3_2735 | 1 | 0.2% |
| UCE | chr3_2742 | 0 | 0.0% |
| UCE | chr3_2999 | 2 | 0.5% |
| UCE | chr3_300 | 0 | 0.0% |
| UCE | chr3_3073 | 3 | 0.7% |
| UCE | chr3_3180 | 1 | 0.3% |
| UCE | chr3_3247 | 2 | 0.5% |
| UCE | chr3_362 | 3 | 0.7% |
| UCE | chr3_3805 | 3 | 0.7% |
| UCE | chr3_3876 | 8 | 1.6% |
| UCE | chr3_457 | 4 | 0.9% |
| UCE | chr3_509 | 4 | 1.0% |
| UCE | chr3_5445 | 2 | 0.5% |
| UCE | chr3_5455 | 1 | 0.3% |
| UCE | chr3_5476 | 0 | 0.0% |
| UCE | chr3_5520 | 0 | 0.0% |
| UCE | chr3_5536 | 5 | 1.5% |
| UCE | chr3_5552 | 1 | 0.2% |
| UCE | chr3_5573 | 6 | 1.4% |
| UCE | chr3_5605 | 4 | 1.0% |
| UCE | chr3_5687 | 2 | 0.5% |
| UCE | chr3_5691 | 7 | 2.0% |
| UCE | chr3_576 | 0 | 0.0% |
| UCE | chr3_5766 | 3 | 0.6% |
| UCE | chr3_5767 | 1 | 0.3% |
| UCE | chr3_5781 | 0 | 0.0% |
| UCE | chr3_5815 | 3 | 0.8% |
| UCE | chr3_5848 | 1 | 0.3% |
| UCE | chr3_5854 | 1 | 0.2% |
| UCE | chr3_5857 | 3 | 0.8% |
| UCE | chr3_5873 | 8 | 2.0% |
| UCE | chr3_5877 | 0 | 0.0% |
| UCE | chr3_5894 | 3 | 0.8% |
| UCE | chr3_5918 | 3 | 0.7% |
| UCE | chr3_5934 | 2 | 0.5% |
| UCE | chr3_6118 | 1 | 0.3% |
| UCE | chr3_6129 | 0 | 0.0% |
| UCE | chr4_10540 | 4 | 0.9% |
| UCE | chr4_10550 | 6 | 1.2% |
| UCE | chr4_10564 | 5 | 1.3% |
| UCE | chr4_11155 | 0 | 0.0% |
| UCE | chr4_11159 | 4 | 1.0% |
| UCE | chr4_13410 | 3 | 0.8% |
| UCE | chr4_13431 | 4 | 1.0% |
| UCE | chr4_13654 | 3 | 0.8% |
| UCE | chr4_15363 | 1 | 0.2% |
| UCE | chr4_15987 | 0 | 0.0% |
| UCE | chr4_17221 | 3 | 0.5% |
| UCE | chr4_17640 | 9 | 2.1% |
| UCE | chr4_6701 | 4 | 1.2% |
| UCE | chr4_6739 | 4 | 1.1% |
| UCE | chr4_7199 | 2 | 0.5% |
| UCE | chr4_7243 | 28 | 6.3% |
| UCE | chr4_7258 | 2 | 0.4% |
| UCE | chr4_7282 | 3 | 0.7% |
| UCE | chr4_7513 | 1 | 0.2% |
| UCE | chr4_7559 | 3 | 0.6% |
| UCE | chr4_7570 | 8 | 1.8% |
| UCE | chr4_9665 | 0 | 0.0% |
| UCE | chr4_9725 | 4 | 0.9% |
| UCE | chr4_9746 | 1 | 0.2% |
| UCE | chr5_10115 | 2 | 0.5% |
| UCE | chr5_10176 | 1 | 0.2% |
| UCE | chr5_10180 | 3 | 0.8% |
| UCE | chr5_10184 | 8 | 2.1% |
| UCE | chr5_10207 | 3 | 0.8% |
| UCE | chr5_10239 | 6 | 1.7% |
| UCE | chr5_10251 | 3 | 0.6% |
| UCE | chr5_10254 | 7 | 2.2% |
| UCE | chr5_10266 | 5 | 1.0% |
| UCE | chr5_10353 | 3 | 0.7% |
| UCE | chr5_10416 | 3 | 0.7% |
| UCE | chr5_10787 | 1 | 0.3% |
| UCE | chr5_10841 | 2 | 0.6% |
| UCE | chr5_10846 | 0 | 0.0% |
| UCE | chr5_10859 | 1 | 0.2% |
| UCE | chr5_10906 | 0 | 0.0% |
| UCE | chr5_11139 | 0 | 0.0% |
| UCE | chr5_11146 | 1 | 0.3% |
| UCE | chr5_11148 | 1 | 0.3% |
| UCE | chr5_11165 | 0 | 0.0% |
| UCE | chr5_11198 | 5 | 1.2% |
| UCE | chr5_11202 | 2 | 0.6% |
| UCE | chr5_11206 | 0 | 0.0% |
| UCE | chr5_11214 | 4 | 0.9% |
| UCE | chr5_11226 | 1 | 0.3% |
| UCE | chr5_11240 | 2 | 0.5% |
| UCE | chr5_11245 | 1 | 0.2% |
| UCE | chr5_11286 | 2 | 0.5% |
| UCE | chr5_11302 | 7 | 1.9% |
| UCE | chr5_11304 | 1 | 0.2% |
| UCE | chr5_11321 | 1 | 0.3% |
| UCE | chr5_11325 | 0 | 0.0% |
| UCE | chr5_11342 | 2 | 0.6% |
| UCE | chr5_11359 | 5 | 1.2% |
| UCE | chr5_11571 | 4 | 1.1% |
| UCE | chr5_11637 | 5 | 1.2% |
| UCE | chr5_11657 | 0 | 0.0% |
| UCE | chr5_11783 | 1 | 0.2% |
| UCE | chr5_11955 | 0 | 0.0% |
| UCE | chr5_12397 | 0 | 0.0% |
| UCE | chr5_12400 | 2 | 0.6% |
| UCE | chr5_12422 | 3 | 0.8% |
| UCE | chr5_12835 | 1 | 0.3% |
| UCE | chr5_13040 | 2 | 0.6% |
| UCE | chr5_13042 | 0 | 0.0% |
| UCE | chr5_14403 | 3 | 0.7% |
| UCE | chr5_14621 | 3 | 0.8% |
| UCE | chr5_14632 | 0 | 0.0% |
| UCE | chr5_14720 | 0 | 0.0% |
| UCE | chr5_14766 | 0 | 0.0% |
| UCE | chr5_14864 | 1 | 0.3% |
| UCE | chr5_14870 | 7 | 1.5% |
| UCE | chr5_14876 | 0 | 0.0% |
| UCE | chr5_14914 | 3 | 0.9% |
| UCE | chr5_15022 | 1 | 0.3% |
| UCE | chr5_15078 | 2 | 0.4% |
| UCE | chr5_1597 | 4 | 1.0% |
| UCE | chr5_1675 | 5 | 1.2% |
| UCE | chr5_1689 | 0 | 0.0% |
| UCE | chr5_1701 | 2 | 0.5% |
| UCE | chr5_1746 | 2 | 0.5% |
| UCE | chr5_1749 | 0 | 0.0% |
| UCE | chr5_1757 | 1 | 0.2% |
| UCE | chr5_1800 | 4 | 0.9% |
| UCE | chr5_1813 | 3 | 0.8% |
| UCE | chr5_1834 | 2 | 0.4% |
| UCE | chr5_1989 | 8 | 2.0% |
| UCE | chr5_3191 | 1 | 0.3% |
| UCE | chr5_3204 | 0 | 0.0% |
| UCE | chr5_3273 | 2 | 0.5% |
| UCE | chr5_3353 | 1 | 0.3% |
| UCE | chr5_3377 | 0 | 0.0% |
| UCE | chr5_3407 | 1 | 0.3% |
| UCE | chr5_3418 | 3 | 0.8% |
| UCE | chr5_4018 | 3 | 0.7% |
| UCE | chr5_5657 | 5 | 1.8% |
| UCE | chr5_8793 | 2 | 0.6% |
| UCE | chr6_6814 | 3 | 0.9% |
| UCE | chr6_8253 | 0 | 0.0% |
| UCE | chr6_8786 | 1 | 0.2% |
| UCE | chr6_8806 | 0 | 0.0% |
| UCE | chr6_8829 | 1 | 0.3% |
| UCE | chr6_9046 | 0 | 0.0% |
| UCE | chr6_9069 | 2 | 0.5% |
| UCE | chr6_9474 | 1 | 0.2% |
| UCE | chr6_9529 | 2 | 0.6% |
| UCE | chr6_9532 | 4 | 0.8% |
| UCE | chr6_9559 | 3 | 0.7% |
| UCE | chr6_9631 | 1 | 0.3% |
| UCE | chr6_9640 | 1 | 0.3% |
| UCE | chr6_9737 | 4 | 1.0% |
| UCE | chr6_9746 | 1 | 0.3% |
| UCE | chr6_9762 | 0 | 0.0% |
| UCE | chr6_9783 | 3 | 0.8% |
| UCE | chr6_9787 | 1 | 0.3% |
| UCE | chr6_9797 | 2 | 0.5% |
| UCE | chr6_9804 | 2 | 0.5% |
| UCE | chr6_9806 | 2 | 0.5% |
| UCE | chr6_9809 | 1 | 0.3% |
| UCE | chr6_9838 | 3 | 0.8% |
| UCE | chr7_10269 | 0 | 0.0% |
| UCE | chr7_10305 | 1 | 0.2% |
| UCE | chr7_10322 | 1 | 0.2% |
| UCE | chr7_10380 | 4 | 1.0% |
| UCE | chr7_10394 | 2 | 0.5% |
| UCE | chr7_10440 | 0 | 0.0% |
| UCE | chr7_10443 | 4 | 0.9% |
| UCE | chr7_10480 | 1 | 0.3% |
| UCE | chr7_10497 | 14 | 3.3% |
| UCE | chr7_10502 | 5 | 1.2% |
| UCE | chr7_10532 | 4 | 0.8% |
| UCE | chr7_10675 | 0 | 0.0% |
| UCE | chr7_10681 | 2 | 0.5% |
| UCE | chr7_10694 | 0 | 0.0% |
| UCE | chr7_1370 | 0 | 0.0% |
| UCE | chr7_1380 | 0 | 0.0% |
| UCE | chr7_6327 | 0 | 0.0% |
| UCE | chr7_6333 | 5 | 1.3% |
| UCE | chr7_6366 | 0 | 0.0% |
| UCE | chr7_9094 | 2 | 0.5% |
| UCE | chr7_9104 | 0 | 0.0% |
| UCE | chr8_3308 | 3 | 0.7% |
| UCE | chr8_4014 | 2 | 0.6% |
| UCE | chr8_4067 | 3 | 0.6% |
| UCE | chr8_4091 | 4 | 1.1% |
| UCE | chr8_4241 | 4 | 1.1% |
| UCE | chr8_4243 | 3 | 0.7% |
| UCE | chr8_4319 | 4 | 1.1% |
| UCE | chr8_4333 | 2 | 0.5% |
| UCE | chr8_4340 | 1 | 0.2% |
| UCE | chr8_4342 | 1 | 0.2% |
| UCE | chr8_4410 | 1 | 0.2% |
| UCE | chr8_6218 | 0 | 0.0% |
| UCE | chr8_6224 | 1 | 0.3% |
| UCE | chr8_6230 | 0 | 0.0% |
| UCE | chr8_6277 | 1 | 0.2% |
| UCE | chr8_6299 | 4 | 1.0% |
| UCE | chr8_6872 | 1 | 0.3% |
| UCE | chr8_7441 | 1 | 0.3% |
| UCE | chr8_7449 | 6 | 1.5% |
| UCE | chr8_7513 | 0 | 0.0% |
| UCE | chr8_7534 | 1 | 0.3% |
| UCE | chr8_8877 | 3 | 0.7% |
| UCE | chr8_8942 | 0 | 0.0% |
| UCE | chr8_9143 | 0 | 0.0% |
| UCE | chr8_9173 | 0 | 0.0% |
| UCE | chr9_1152 | 1 | 0.2% |
| UCE | chr9_1164 | 3 | 0.7% |
| UCE | chr9_1169 | 0 | 0.0% |
| UCE | chr9_1191 | 1 | 0.3% |
| UCE | chr9_1949 | 1 | 0.3% |
| UCE | chr9_2499 | 5 | 1.2% |
| UCE | chr9_3289 | 7 | 1.7% |
| UCE | chr9_3633 | 3 | 0.7% |
| UCE | chr9_5181 | 4 | 1.1% |
| UCE | chr9_5205 | 1 | 0.2% |
| UCE | chr9_5220 | 0 | 0.0% |
| UCE | chr9_5246 | 1 | 0.2% |
| UCE | chr9_6320 | 5 | 1.2% |
| UCE | chr9_6322 | 5 | 1.3% |
| UCE | chr9_6325 | 7 | 1.7% |
| UCE | chr9_6414 | 1 | 0.2% |
| UCE | chr9_7171 | 1 | 0.2% |
| UCE | chr9_7189 | 0 | 0.0% |
| UCE | chr9_7434 | 4 | 1.0% |
| UCE | chrun_random_11933 | 1 | 0.2% |
| UCE | chrun_random_3551 | 0 | 0.0% |
| UCE | chrun_random_7197 | 1 | 0.2% |
| UCE | chrz_11272 | 1 | 0.3% |
| UCE | chrz_11397 | 3 | 0.7% |
| UCE | chrz_11457 | 0 | 0.0% |
| UCE | chrz_11465 | 1 | 0.2% |
| UCE | chrz_11477 | 4 | 1.1% |
| UCE | chrz_11491 | 0 | 0.0% |
| UCE | chrz_11540 | 10 | 2.7% |
| UCE | chrz_11557 | 3 | 0.7% |
| UCE | chrz_11584 | 4 | 1.0% |
| UCE | chrz_11684 | 4 | 0.9% |
| UCE | chrz_4313 | 0 | 0.0% |
| UCE | chrz_467 | 2 | 0.5% |
| UCE | chrz_4740 | 1 | 0.3% |
| UCE | chrz_4747 | 5 | 1.4% |
| UCE | chrz_4759 | 1 | 0.2% |
| UCE | chrz_4763 | 2 | 0.5% |
| UCE | chrz_4772 | 6 | 1.1% |
| UCE | chrz_4782 | 4 | 1.0% |
| UCE | chrz_4787 | 2 | 0.4% |
| UCE | chrz_4794 | 3 | 0.8% |
| UCE | chrz_4816 | 2 | 0.6% |
| UCE | chrz_4832 | 1 | 0.2% |
| UCE | chrz_4838 | 2 | 0.7% |
| UCE | chrz_4841 | 3 | 0.7% |
| UCE | chrz_5495 | 10 | 2.0% |
| UCE | chrz_5501 | 1 | 0.2% |
| UCE | chrz_6357 | 3 | 0.8% |
| UCE | chrz_6396 | 8 | 2.1% |
| UCE | chrz_646 | 2 | 0.6% |
| UCE | chrz_6575 | 8 | 1.8% |
| UCE | chrz_6612 | 1 | 0.2% |
| UCE | chrz_6686 | 0 | 0.0% |
| UCE | chrz_6690 | 4 | 1.0% |
| UCE | chrz_6703 | 10 | 2.3% |
| UCE | chrz_6778 | 1 | 0.2% |
| UCE | chrz_6799 | 0 | 0.0% |
| UCE | chrz_7406 | 5 | 1.2% |
| UCE | chrz_7416 | 2 | 0.5% |
| UCE | chrz_7824 | 2 | 0.5% |
| UCE | chrz_7889 | 3 | 0.6% |
| UCE | chrz_7923 | 4 | 1.1% |
| UCE | chrz_7943 | 18 | 4.2% |
| UCE | chrz_7982 | 3 | 0.7% |
| UCE | chrz_7997 | 0 | 0.0% |
| UCE | chrz_8024 | 0 | 0.0% |
| Protein-coding | ADNP | 3 | 0.8% |
| Protein-coding | AKAP9 | 5 | 1.3% |
| Protein-coding | ANR | 7 | 2.1% |
| Protein-coding | BACH1 | 9 | 2.4% |
| Protein-coding | BDNF | 2 | 0.5% |
| Protein-coding | BHLHB2 | 4 | 1.0% |
| Protein-coding | BMP2 | 10 | 2.3% |
| Protein-coding | CAND1 | 8 | 1.8% |
| Protein-coding | CARD4 | 11 | 2.8% |
| Protein-coding | CILP | 2 | 0.6% |
| Protein-coding | CXCR4 | 11 | 2.6% |
| Protein-coding | DLL1 | 6 | 1.3% |
| Protein-coding | ECEL | 7 | 2.0% |
| Protein-coding | ENC6 | 9 | 1.8% |
| Protein-coding | FSHR | 5 | 1.2% |
| Protein-coding | FSTL5 | 3 | 0.6% |
| Protein-coding | GALR1 | 2 | 0.5% |
| Protein-coding | GHSR | 2 | 0.5% |
| Protein-coding | GPR37 | 7 | 1.3% |
| Protein-coding | HLCS | 6 | 2.0% |
| Protein-coding | INHIBA | 4 | 0.8% |
| Protein-coding | LRRN1 | 10 | 2.3% |
| Protein-coding | LZTSS1 | 2 | 0.6% |
| Protein-coding | MKL1 | 3 | 0.7% |
| Protein-coding | MLL3 | 23 | 6.3% |
| Protein-coding | MSH6 | 32 | 7.8% |
| Protein-coding | NGFB | 10 | 2.3% |
| Protein-coding | NKTR | 16 | 4.7% |
| Protein-coding | NTF3 | 7 | 1.9% |
| Protein-coding | PNN | 16 | 4.3% |
| Protein-coding | PRLR | 9 | 2.4% |
| Protein-coding | PTGER4 | 8 | 1.9% |
| Protein-coding | PTPN | 15 | 3.8% |
| Protein-coding | R35 | 3 | 0.8% |
| Protein-coding | RAG1 | 7 | 1.8% |
| Protein-coding | SINAIP | 3 | 0.9% |
| Protein-coding | SLC30A1 | 17 | 3.9% |
| Protein-coding | SLC8A1 | 9 | 2.1% |
| Protein-coding | SLC8A3 | 2 | 0.5% |
| Protein-coding | TRAF6 | 12 | 3.5% |
| Protein-coding | VCPIP1 | 9 | 1.9% |
| Protein-coding | ZEB2 | 4 | 1.1% |
| Protein-coding | ZFP36L1 | 12 | 2.8% |
