# Supplementary material for: Phylogenomic analysis of the Chilean clade of Liolaemus lizards (Squamata: Liolaemidae) based on sequence capture data
Source: PeerJ. 2017 Oct 26;5:e3941. doi: 10.7717/peerj.3941 (PMC5660876; doi:10.7717/peerj.3941)
Supplement: Table S5 [file peerj-05-3941-s005.docx]

| Marker | IQ-TREE | Jmodeltest |
| --- | --- | --- |
| ADNP | JC | JC |
| AKAP9 | HKY | HKY |
| ANR | K80+I | K80+I |
| BACH1 | K3P+G4 | K80 |
| BDNF | K80+I | K80 |
| BHLHB2 | K80+I | K80 |
| BMP2 | K80+I | K80+I |
| CAND1 | K80+I | K80+I |
| CARD4 | K80+I | K80+I |
| CILP | JC+I | K80 |
| CXCR4 | K80+I | K80+I |
| DLL1 | K80+I | K80 |
| ECEL | TN+I | HKY+I |
| ENC6 | K80+I | K80 |
| FSHR | K80+I | K80+I |
| FSTL5 | HKY | HKY |
| GALR1 | HKY+I | HKY+I |
| GHSR | K80 | K80 |
| GPR37 | K80+I | K80 |
| HLCS | HKY+I | HKY+I |
| INHIBA | K80 | K80 |
| LRRN1 | HKY+I | HKY+I |
| LZTSS1 | HKY+I | HKY+I |
| MKL1 | K80+I | K80+I |
| MLL3 | HKY | HKY |
| MSH6 | K80 | K80 |
| NGFB | K80+I | K80+I |
| NKTR | HKY+G4 | HKY |
| NTF3 | K80 | K80 |
| PNN | HKY+G4 | HKY+G |
| PRLR | K80 | K80 |
| PTGER4 | TN+I | HKY+I |
| PTPN | K80+I | K80+I |
| R35 | HKY+I | HKY |
| RAG1 | K80+I | K80+I |
| SINAIP | JC+I | JC |
| SLC30A1 | K80+I | K80+I |
| SLC8A1 | K80+I | K80+I |
| SLC8A3 | K80+I | K80 |
| TRAF6 | K80+I | K80 |
| VCPIP1 | HKY | HKY |
| ZEB2 | K80+I | K80 |
| ZFP36L1 | HKY | HKY |
| chr12_1169 | JC | JC |
| chr12_1475 | K3Pu | HKY |
| chr12_2213 | HKY+I | HKY |
| chr12_2426 | F81 | F81 |
| chr12_3124 | K80 | K80 |
| chr12_3154 | F81 | F81 |
| chr12_3865 | JC | JC |
| chr12_5665 | K80 | K80 |
| chr12_5671 | F81 | F81 |
| chr12_5730 | F81 | F81 |
| chr12_5739 | F81 | F81 |
| chr12_5828 | F81 | F81 |
| chr12_5837 | HKY | HKY |
| chr12_5840 | F81 | HKY |
| chr12_5851 | F81 | F81 |
| chr12_5878 | HKY+I | HKY |
| chr12_5895 | HKY+I | F81 |
| chr12_5903 | HKY | HKY |
| chr12_5908 | F81 | F81 |
| chr12_5912 | TPM3u+I | F81+I+G |
| chr12_5949 | K80+I | K80 |
| chr12_5969 | F81+I | F81+G |
| chr13_1225 | F81 | F81 |
| chr13_4268 | F81 | F81 |
| chr13_5059 | F81+I | F81 |
| chr13_5324 | F81 | F81 |
| chr13_710 | HKY+I | HKY |
| chr13_720 | F81 | F81 |
| chr13_726 | F81 | F81 |
| chr18_1422 | F81+I | F81 |
| chr18_4493 | F81 | F81 |
| chr1_10949 | K80 | K80 |
| chr1_13047 | JC+I | JC |
| chr1_13198 | JC+I | JC |
| chr1_13698 | JC | JC |
| chr1_1378 | F81+R2 | F81 |
| chr1_1418 | F81 | F81 |
| chr1_14389 | F81 | F81 |
| chr1_14672 | HKY+I | HKY+I |
| chr1_15412 | F81 | F81 |
| chr1_15480 | F81 | F81 |
| chr1_15632 | HKY+I | K80 |
| chr1_16199 | HKY | HKY |
| chr1_18915 | K80+I | K80 |
| chr1_19202 | F81 | F81 |
| chr1_19246 | F81 | HKY |
| chr1_19292 | F81 | F81 |
| chr1_19426 | K80 | K80 |
| chr1_19885 | K80+I | K80 |
| chr1_21570 | F81 | F81 |
| chr1_22432 | K80 | JC |
| chr1_23573 | JC+I | JC+G |
| chr1_24625 | F81 | F81 |
| chr1_24640 | K80+I | K80 |
| chr1_24644 | F81 | F81 |
| chr1_24817 | F81 | F81 |
| chr1_25630 | HKY | HKY |
| chr1_25670 | F81 | F81 |
| chr1_25675 | F81 | F81 |
| chr1_25680 | F81 | F81 |
| chr1_25692 | TN | HKY |
| chr1_25699 | F81 | F81 |
| chr1_25705 | HKY+I | HKY |
| chr1_26035 | F81+I | F81 |
| chr1_27509 | HKY+I | HKY+I |
| chr1_27552 | TN | HKY |
| chr1_2930 | K80 | K80 |
| chr1_29790 | F81 | F81 |
| chr1_29835 | F81 | F81 |
| chr1_29841 | F81 | F81 |
| chr1_29894 | HKY+I | HKY |
| chr1_29912 | JC | JC |
| chr1_30195 | F81+I | F81 |
| chr1_30635 | F81 | F81 |
| chr1_31673 | F81 | F81 |
| chr1_31677 | F81+I | F81 |
| chr1_31709 | HKY | HKY |
| chr1_31743 | HKY | HKY |
| chr1_31749 | F81 | F81 |
| chr1_31783 | HKY+I | HKY |
| chr1_32194 | K80 | K80 |
| chr1_32208 | F81 | F81 |
| chr1_32232 | HKY+I | F81 |
| chr1_32234 | F81 | F81 |
| chr1_32266 | HKY+I | HKY+I |
| chr1_32286 | HKY+I | F81 |
| chr1_32322 | F81 | F81 |
| chr1_32333 | HKY+I | HKY |
| chr1_32337 | F81+I | F81 |
| chr1_32356 | F81+I | F81 |
| chr1_32365 | F81 | F81 |
| chr1_32370 | F81 | F81 |
| chr1_32378 | TPM2u+I | F81 |
| chr1_32429 | HKY+I | F81 |
| chr1_32443 | F81 | HKY |
| chr1_32461 | HKY+I | HKY+I |
| chr1_33834 | K80+I | K80 |
| chr1_34776 | HKY+I | HKY+I |
| chr1_3857 | HKY | HKY |
| chr1_4680 | HKY+I | HKY |
| chr1_5277 | JC | JC |
| chr1_5279 | F81 | F81 |
| chr1_5288 | F81 | F81 |
| chr1_5301 | F81+I | F81 |
| chr1_5319 | JC | JC |
| chr1_5334 | HKY | HKY |
| chr1_5365 | F81+I | F81 |
| chr1_5379 | F81 | F81 |
| chr1_5409 | F81 | F81 |
| chr1_5426 | F81 | F81 |
| chr1_5466 | HKY | HKY |
| chr1_5470 | F81 | F81 |
| chr1_5474 | F81+I | F81 |
| chr1_5479 | F81 | F81 |
| chr1_5492 | F81 | F81 |
| chr1_8658 | HKY+I | HKY |
| chr1_8991 | HKY+I | HKY |
| chr20_1391 | HKY | HKY |
| chr20_253 | HKY | HKY |
| chr20_3629 | K80+I | K80+I |
| chr26_2189 | HKY+I | HKY |
| chr26_2766 | F81 | F81 |
| chr26_2850 | F81 | F81 |
| chr2_11187 | TPM3+I | F81 |
| chr2_11494 | HKY+I | HKY |
| chr2_11510 | HKY | HKY |
| chr2_11732 | HKY | HKY |
| chr2_11743 | TNe+I | HKY |
| chr2_11785 | K80+I | K80 |
| chr2_11789 | F81 | F81 |
| chr2_11804 | HKY | HKY |
| chr2_12928 | JC+I | JC |
| chr2_12992 | F81 | F81 |
| chr2_12994 | HKY | HKY |
| chr2_13030 | HKY | HKY |
| chr2_13032 | F81 | F81 |
| chr2_13034 | HKY+I | HKY |
| chr2_13064 | F81 | F81 |
| chr2_13460 | JC+I | JC+I+G |
| chr2_13502 | HKY+I | HKY+I |
| chr2_1647 | F81 | F81 |
| chr2_17005 | F81 | F81 |
| chr2_17019 | F81 | F81 |
| chr2_17532 | HKY+I | HKY |
| chr2_18468 | TPM3u+I | HKY |
| chr2_18477 | JC+I | JC |
| chr2_18557 | HKY | F81 |
| chr2_18578 | F81 | F81 |
| chr2_18589 | JC | JC |
| chr2_18608 | F81 | F81 |
| chr2_18614 | HKY+R2 | HKY+I |
| chr2_18619 | K80+I | JC |
| chr2_18662 | F81 | F81 |
| chr2_18677 | JC | JC |
| chr2_18686 | JC | JC |
| chr2_18714 | JC | JC |
| chr2_18743 | F81 | F81 |
| chr2_1916 | JC | JC |
| chr2_20477 | F81+I | F81 |
| chr2_21229 | F81 | F81 |
| chr2_21265 | F81 | F81 |
| chr2_21284 | F81 | F81 |
| chr2_21308 | JC | JC |
| chr2_21320 | F81+I | F81 |
| chr2_21344 | F81 | F81 |
| chr2_21358 | HKY+I | HKY+I |
| chr2_21401 | F81 | F81 |
| chr2_21445 | K80 | K80 |
| chr2_2239 | K3P+I | JC |
| chr2_23113 | TN | HKY |
| chr2_23160 | HKY | HKY |
| chr2_23221 | F81+I | HKY |
| chr2_23596 | F81 | F81 |
| chr2_23621 | HKY+I | HKY+I |
| chr2_23635 | JC | JC |
| chr2_23648 | TNe+I | K80+I |
| chr2_23668 | F81 | F81 |
| chr2_24173 | F81+I | F81 |
| chr2_24655 | HKY+I | HKY |
| chr2_24672 | K3Pu+I | HKY+I |
| chr2_24684 | F81 | F81 |
| chr2_24697 | HKY+I | F81 |
| chr2_24704 | F81+I | F81 |
| chr2_24800 | HKY | HKY |
| chr2_24815 | JC | JC |
| chr2_24827 | HKY | HKY |
| chr2_24841 | F81+I | F81 |
| chr2_24859 | K80+I | K80+I |
| chr2_24876 | F81 | F81 |
| chr2_24879 | HKY | HKY |
| chr2_24910 | JC | JC |
| chr2_25833 | F81+I | F81+G |
| chr2_25851 | JC+I | JC+G |
| chr2_27241 | F81+I | F81 |
| chr2_27258 | F81 | F81 |
| chr2_27261 | F81 | F81 |
| chr2_27280 | F81 | F81 |
| chr2_27294 | F81 | F81 |
| chr2_27313 | F81 | F81 |
| chr2_27968 | F81 | F81 |
| chr2_29406 | F81+I | F81 |
| chr2_4395 | F81 | F81 |
| chr2_5491 | K80 | K80 |
| chr2_5499 | JC | JC |
| chr2_5526 | JC | JC |
| chr2_5991 | F81 | F81 |
| chr2_6341 | JC | JC |
| chr2_6444 | JC | JC |
| chr2_6685 | F81 | F81 |
| chr2_6737 | F81 | F81 |
| chr2_6787 | F81 | F81 |
| chr2_7409 | F81 | F81 |
| chr2_7420 | HKY+I | HKY |
| chr2_7927 | F81 | F81 |
| chr2_7945 | F81+I | F81 |
| chr2_7954 | F81 | F81 |
| chr2_8583 | HKY+I | HKY+I |
| chr2_8589 | F81 | F81 |
| chr2_8590 | F81 | F81 |
| chr2_8600 | F81+I | F81 |
| chr2_8609 | F81 | F81 |
| chr2_8620 | HKY | HKY |
| chr2_8629 | HKY+I | HKY+I |
| chr2_8651 | JC+I | JC |
| chr2_8655 | HKY | HKY |
| chr2_8677 | HKY+I | HKY |
| chr2_8688 | JC | JC |
| chr2_8698 | HKY+I | HKY |
| chr2_8747 | F81+I | F81 |
| chr2_8754 | F81 | F81 |
| chr3_11795 | F81+I | F81 |
| chr3_11879 | K80+I | K80 |
| chr3_1282 | F81+I | F81 |
| chr3_1300 | F81 | F81 |
| chr3_13359 | HKY | HKY |
| chr3_13404 | HKY | F81 |
| chr3_16820 | F81+I | F81 |
| chr3_16833 | HKY+I | HKY+I |
| chr3_17448 | HKY+I | HKY |
| chr3_17607 | JC+I | JC |
| chr3_17623 | F81 | F81 |
| chr3_17699 | HKY+I | HKY |
| chr3_17721 | JC | JC |
| chr3_17747 | K80+I | JC |
| chr3_17769 | JC | JC |
| chr3_17781 | F81 | F81 |
| chr3_17860 | JC | JC |
| chr3_17890 | F81 | F81 |
| chr3_17991 | HKY | HKY |
| chr3_18203 | F81 | F81 |
| chr3_18256 | F81 | F81 |
| chr3_18306 | JC+I | JC |
| chr3_19568 | F81 | F81 |
| chr3_19997 | F81 | F81 |
| chr3_20013 | TN+I | HKY |
| chr3_21510 | F81+I | HKY |
| chr3_21949 | K80+I | K80+I |
| chr3_22024 | K80+I | JC |
| chr3_23724 | F81 | F81 |
| chr3_24903 | JC+I | JC |
| chr3_24963 | K80 | K80 |
| chr3_25095 | F81+I | F81+G |
| chr3_2698 | HKY+I | HKY |
| chr3_2723 | F81 | F81 |
| chr3_2735 | F81+I | F81 |
| chr3_2742 | F81 | F81 |
| chr3_2999 | F81 | F81 |
| chr3_300 | HKY | HKY |
| chr3_3073 | JC+I | JC+I+G |
| chr3_3180 | JC | JC |
| chr3_3247 | F81+I | F81 |
| chr3_362 | F81 | F81 |
| chr3_3805 | JC | JC |
| chr3_3876 | TPM2u+I | HKY |
| chr3_457 | TNe+I | K80 |
| chr3_509 | F81 | F81 |
| chr3_5445 | HKY | F81 |
| chr3_5455 | JC | JC |
| chr3_5476 | F81 | F81 |
| chr3_5520 | F81 | F81 |
| chr3_5536 | F81 | F81 |
| chr3_5552 | HKY+I | HKY |
| chr3_5573 | TN | F81 |
| chr3_5605 | HKY+I | HKY |
| chr3_5687 | HKY | HKY |
| chr3_5691 | HKY | HKY |
| chr3_576 | F81 | F81 |
| chr3_5766 | HKY+I | F81 |
| chr3_5767 | JC | JC |
| chr3_5781 | JC | JC |
| chr3_5815 | HKY+I | HKY |
| chr3_5848 | HKY | HKY |
| chr3_5854 | HKY | HKY |
| chr3_5857 | HKY | HKY |
| chr3_5873 | K80+I | K80 |
| chr3_5877 | JC | JC |
| chr3_5894 | F81 | F81 |
| chr3_5918 | JC | F81 |
| chr3_5934 | HKY+I | F81 |
| chr3_6118 | K80 | K80 |
| chr3_6129 | JC | JC |
| chr4_10540 | K80+I | K80 |
| chr4_10550 | K80+I | K80 |
| chr4_10564 | HKY+I | F81 |
| chr4_11155 | F81 | F81 |
| chr4_11159 | JC+I | JC |
| chr4_13410 | HKY+I | HKY |
| chr4_13431 | K80+I | K80+I |
| chr4_13654 | F81+I | F81 |
| chr4_15363 | F81+I | F81 |
| chr4_15987 | F81 | F81 |
| chr4_17221 | JC+I | HKY+I |
| chr4_17640 | HKY | HKY |
| chr4_6701 | HKY | HKY |
| chr4_6739 | HKY+I | HKY |
| chr4_7199 | HKY | HKY |
| chr4_7243 | K80+I | K80+I |
| chr4_7258 | F81+I | HKY |
| chr4_7282 | F81 | HKY |
| chr4_7513 | JC | JC |
| chr4_7559 | TNe+I | K80 |
| chr4_7570 | K80+I | K80 |
| chr4_9665 | F81 | F81 |
| chr4_9725 | HKY+I | HKY |
| chr4_9746 | K80 | K80 |
| chr5_10115 | F81 | F81 |
| chr5_10176 | JC | JC |
| chr5_10180 | K80+I | K80 |
| chr5_10184 | HKY+I | HKY |
| chr5_10207 | HKY+I | F81 |
| chr5_10239 | K80+I | K80+I |
| chr5_10251 | TPM2u | F81 |
| chr5_10254 | HKY+I | HKY |
| chr5_10266 | HKY+I | HKY |
| chr5_10353 | F81 | F81 |
| chr5_10416 | HKY+I | HKY |
| chr5_10787 | JC | JC |
| chr5_10841 | F81 | F81 |
| chr5_10846 | F81 | F81 |
| chr5_10859 | F81 | F81 |
| chr5_10906 | F81 | F81 |
| chr5_11139 | F81 | F81 |
| chr5_11146 | F81 | F81 |
| chr5_11148 | F81 | F81 |
| chr5_11165 | HKY | HKY |
| chr5_11198 | HKY+I | HKY |
| chr5_11202 | HKY | HKY |
| chr5_11206 | F81 | F81 |
| chr5_11214 | JC+I | F81 |
| chr5_11226 | F81 | F81 |
| chr5_11240 | F81 | F81 |
| chr5_11245 | F81 | F81 |
| chr5_11286 | HKY | F81 |
| chr5_11302 | F81+I | F81 |
| chr5_11304 | K80 | K80 |
| chr5_11321 | F81 | F81 |
| chr5_11325 | F81 | F81 |
| chr5_11342 | F81 | F81 |
| chr5_11359 | HKY+I | F81 |
| chr5_11571 | F81 | F81 |
| chr5_11637 | K80 | K80 |
| chr5_11657 | F81 | F81 |
| chr5_11783 | HKY | HKY |
| chr5_11955 | F81 | F81 |
| chr5_12397 | F81 | F81 |
| chr5_12400 | JC | JC |
| chr5_12422 | K80+I | K80 |
| chr5_12835 | F81 | F81 |
| chr5_13040 | F81+I | F81 |
| chr5_13042 | JC | JC |
| chr5_14403 | HKY+I | F81 |
| chr5_14621 | HKY+I | HKY |
| chr5_14632 | F81 | F81 |
| chr5_14720 | F81 | F81 |
| chr5_14766 | JC | JC |
| chr5_14864 | HKY | HKY |
| chr5_14870 | K80+I | K80 |
| chr5_14876 | F81 | F81 |
| chr5_14914 | K80+I | K80 |
| chr5_15022 | K80 | K80 |
| chr5_15078 | HKY+I | HKY |
| chr5_1597 | K80+I | K80 |
| chr5_1675 | K80+I | K80 |
| chr5_1689 | JC | JC |
| chr5_1701 | JC+I | JC+I |
| chr5_1746 | HKY+R2 | HKY |
| chr5_1749 | K80 | K80 |
| chr5_1757 | K80 | JC |
| chr5_1800 | F81 | F81 |
| chr5_1813 | F81 | F81 |
| chr5_1834 | F81+I | HKY |
| chr5_1989 | HKY+I | HKY+I |
| chr5_3191 | F81 | F81 |
| chr5_3204 | F81 | F81 |
| chr5_3273 | HKY | HKY |
| chr5_3353 | F81+I | F81 |
| chr5_3377 | K80 | K80 |
| chr5_3407 | TN | HKY |
| chr5_3418 | F81 | F81 |
| chr5_4018 | K80+I | JC |
| chr5_5657 | F81+I | F81 |
| chr5_8793 | F81+I | F81 |
| chr6_6814 | F81 | F81 |
| chr6_8253 | F81 | F81 |
| chr6_8786 | F81+I | F81 |
| chr6_8806 | HKY | HKY |
| chr6_8829 | F81+I | F81 |
| chr6_9046 | F81 | F81 |
| chr6_9069 | F81 | F81 |
| chr6_9474 | K80+I | K80 |
| chr6_9529 | F81+I | F81 |
| chr6_9532 | JC+I | JC |
| chr6_9559 | HKY+I | HKY |
| chr6_9631 | F81 | F81 |
| chr6_9640 | F81+I | F81 |
| chr6_9737 | F81+I | F81 |
| chr6_9746 | F81+I | F81 |
| chr6_9762 | F81 | F81 |
| chr6_9783 | F81 | F81 |
| chr6_9787 | F81+I | F81 |
| chr6_9797 | HKY+I | HKY |
| chr6_9804 | K80+I | K80 |
| chr6_9806 | JC | JC |
| chr6_9809 | JC+I | JC |
| chr6_9838 | HKY+I | HKY |
| chr7_10269 | F81 | F81 |
| chr7_10305 | F81+I | F81 |
| chr7_10322 | F81 | F81 |
| chr7_10380 | F81 | F81 |
| chr7_10394 | F81 | F81 |
| chr7_10440 | F81 | F81 |
| chr7_10443 | F81+I | F81 |
| chr7_10480 | F81 | F81 |
| chr7_10497 | F81+I | F81+G |
| chr7_10502 | K80+I | K80 |
| chr7_10532 | F81+I | F81 |
| chr7_10675 | F81 | F81 |
| chr7_10681 | JC+I | JC |
| chr7_10694 | F81 | F81 |
| chr7_1370 | F81 | F81 |
| chr7_1380 | F81 | F81 |
| chr7_6327 | K80 | K80 |
| chr7_6333 | HKY+I | HKY |
| chr7_6366 | JC | JC |
| chr7_9094 | K80+I | JC |
| chr7_9104 | F81 | F81 |
| chr8_3308 | F81+I | F81 |
| chr8_4014 | K80+I | K80 |
| chr8_4067 | F81+I | F81 |
| chr8_4091 | HKY | HKY |
| chr8_4241 | TNe+I | K80 |
| chr8_4243 | HKY | HKY |
| chr8_4319 | F81 | F81 |
| chr8_4333 | K80+I | K80 |
| chr8_4340 | F81 | F81 |
| chr8_4342 | JC | JC |
| chr8_4410 | F81 | F81 |
| chr8_6218 | F81 | F81 |
| chr8_6224 | JC | JC |
| chr8_6230 | HKY | HKY |
| chr8_6277 | K80+I | K80 |
| chr8_6299 | JC+I | JC |
| chr8_6872 | F81 | F81 |
| chr8_7441 | F81 | F81 |
| chr8_7449 | K80+I | K80 |
| chr8_7513 | F81 | F81 |
| chr8_7534 | F81 | F81 |
| chr8_8877 | HKY+I | HKY |
| chr8_8942 | F81 | F81 |
| chr8_9143 | F81 | F81 |
| chr8_9173 | F81 | F81 |
| chr9_1152 | F81 | F81 |
| chr9_1164 | JC | JC |
| chr9_1169 | F81 | F81 |
| chr9_1191 | F81 | F81 |
| chr9_1949 | F81 | F81 |
| chr9_2499 | HKY | HKY |
| chr9_3289 | HKY+I | HKY |
| chr9_3633 | F81 | F81 |
| chr9_5181 | HKY+I | HKY |
| chr9_5205 | F81 | F81 |
| chr9_5220 | F81 | F81 |
| chr9_5246 | HKY+I | HKY |
| chr9_6320 | K80+I | K80 |
| chr9_6322 | F81 | F81 |
| chr9_6325 | HKY+I | HKY+I |
| chr9_6414 | JC+I | JC |
| chr9_7171 | JC+I | JC |
| chr9_7189 | F81 | F81 |
| chr9_7434 | HKY | HKY |
| chrun_random_11933 | F81+I | HKY |
| chrun_random_3551 | F81 | F81 |
| chrun_random_7197 | F81 | F81 |
| chrz_11272 | F81 | F81 |
| chrz_11397 | F81+I | F81+I |
| chrz_11457 | F81 | F81 |
| chrz_11465 | F81 | F81 |
| chrz_11477 | HKY | HKY |
| chrz_11491 | JC | JC |
| chrz_11540 | HKY+I | HKY |
| chrz_11557 | F81+I | F81 |
| chrz_11584 | TPM2+I | F81 |
| chrz_11684 | HKY+I | HKY |
| chrz_4313 | F81 | F81 |
| chrz_467 | HKY | HKY |
| chrz_4740 | F81 | F81 |
| chrz_4747 | F81+I | F81 |
| chrz_4759 | HKY+I | F81 |
| chrz_4763 | JC | JC |
| chrz_4772 | K80+I | K80+I |
| chrz_4782 | F81+I | F81 |
| chrz_4787 | F81 | F81 |
| chrz_4794 | F81+I | F81 |
| chrz_4816 | TN+I | F81 |
| chrz_4832 | F81+I | F81 |
| chrz_4838 | HKY+I | F81 |
| chrz_4841 | HKY+I | F81 |
| chrz_5495 | JC+I | K80+I |
| chrz_5501 | F81+I | F81 |
| chrz_6357 | F81 | JC |
| chrz_6396 | K3P+I | K80+I |
| chrz_646 | HKY | HKY |
| chrz_6575 | HKY+I | HKY+I |
| chrz_6612 | F81 | HKY |
| chrz_6686 | JC | JC |
| chrz_6690 | JC+I | JC |
| chrz_6703 | K80+I | K80+I |
| chrz_6778 | HKY | HKY |
| chrz_6799 | JC | JC |
| chrz_7406 | HKY+I | HKY |
| chrz_7416 | K80+I | HKY |
| chrz_7824 | JC+I | F81 |
| chrz_7889 | JC+I | JC |
| chrz_7923 | F81+I | F81 |
| chrz_7943 | TNe+I | K80+I |
| chrz_7982 | HKY+I | F81 |
| chrz_7997 | F81 | F81 |
| chrz_8024 | F81 | F81 |
