# Supplementary material for: Phylogenomic analysis of the Chilean clade of Liolaemus lizards (Squamata: Liolaemidae) based on sequence capture data
Source: PeerJ. 2017 Oct 26;5:e3941. doi: 10.7717/peerj.3941 (PMC5660876; doi:10.7717/peerj.3941)
Supplement: Table S6 — Bold numbers indicate high bootstrap support values (>70) and asterisks indicate that L. t. tenuis belong to that particular clade. [file peerj-05-3941-s006.docx]

| Clade name | Clade relationships | IQ-TREE | | SVD quartets | | ASTRAL-II | |
| --- | --- | --- | --- | --- | --- | --- | --- |
|  |  | With *L. t. tenuis* | Without *L. t. tenuis* | With *L. t. tenuis* | Without *L. t. tenuis* | With *L. t. tenuis* | Without *L. t. tenuis* |
| A | (*L. t. punctatissimu*s, *L. sp*.) | **100** | **100** | **97** | **99** | **99** | **99** |
| B | (*L. fuscus*, *L. nitidus*, *L. monticola*, *L. nigroviridis*) | **99** | **100** | **99** | **100** | **88*** | **99** |
| C | (*L. zapallarensis*, *L. nigromaculatu*s) | **100** | **100** | **98** | **98.5** | **100** | **100** |
| D | (*L. platei*, *L. velosoi*) | **100** | **100** | **96** | **100** | **100** | **100** |
| E | (C+D) | **100** | **100** | **94** | **100** | **98** | **98** |
| F | (B+E) | **99** | **100** | **91.5** | **100** | 63 | 65 |
| G | (A+F) | **99** | **100** | 61 | **75** | **99** | **86** |
| H | (*L. pictus*, *L. paulinae*) | **100** | **100** | **100** | **100** | **100** | **100** |
| I | (H+F) | **98** | **100** | 33 | **100** | **100** | **100** |
| J | (*L. atacamensis*, *L. isabelae*) | **93*** | **100** | **99** | **99** | **92** | **93** |
