# Supplementary figures and images for: Phylogenomic analysis of the Chilean clade of Liolaemus lizards (Squamata: Liolaemidae) based on sequence capture data
Source: PeerJ. 2017 Oct 26;5:e3941. doi: 10.7717/peerj.3941 (PMC5660876; doi:10.7717/peerj.3941)

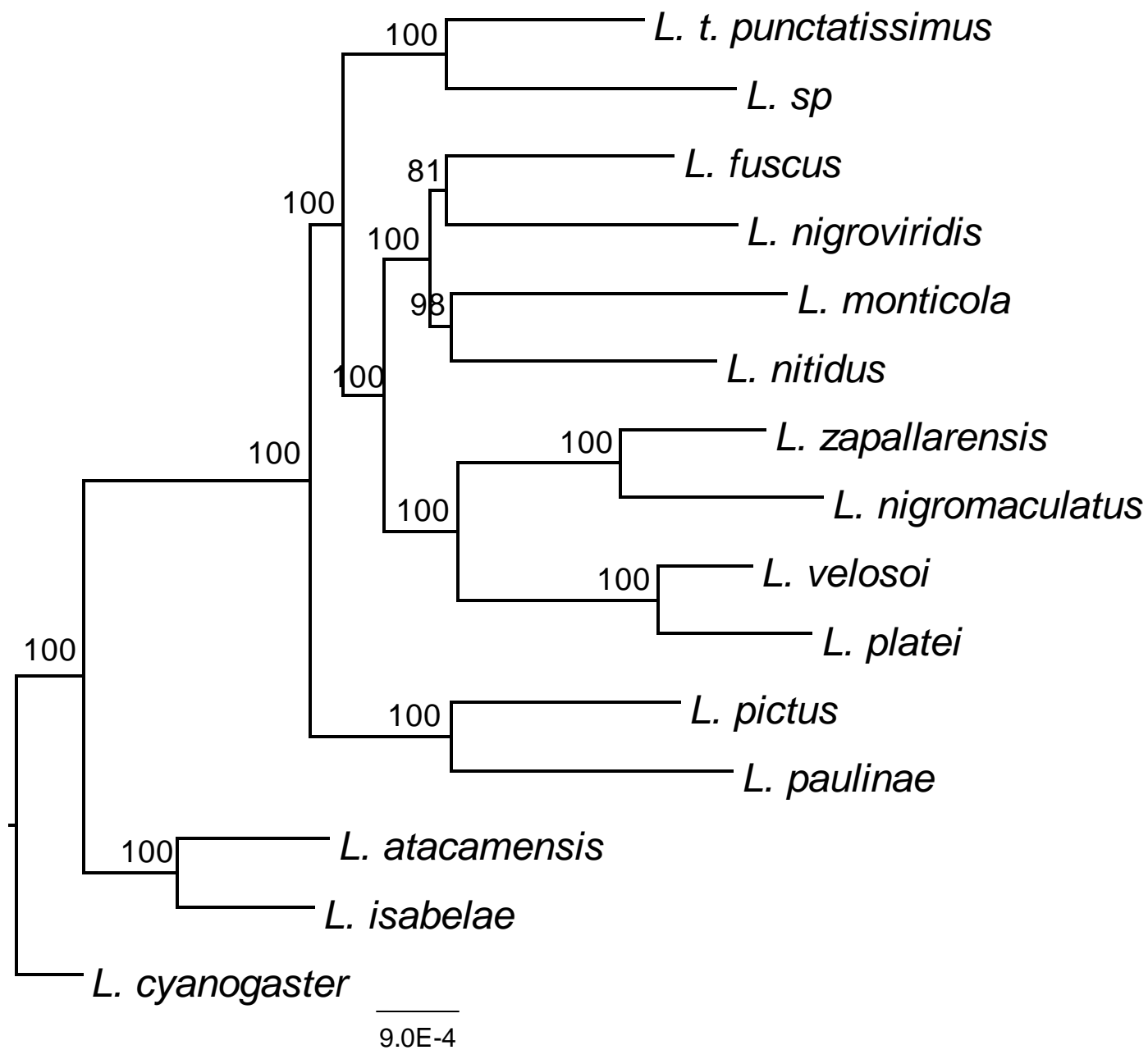

Supplement: Figure S1 — Values next to nodes indicate bootstrap support. [file peerj-05-3941-s007.pdf]

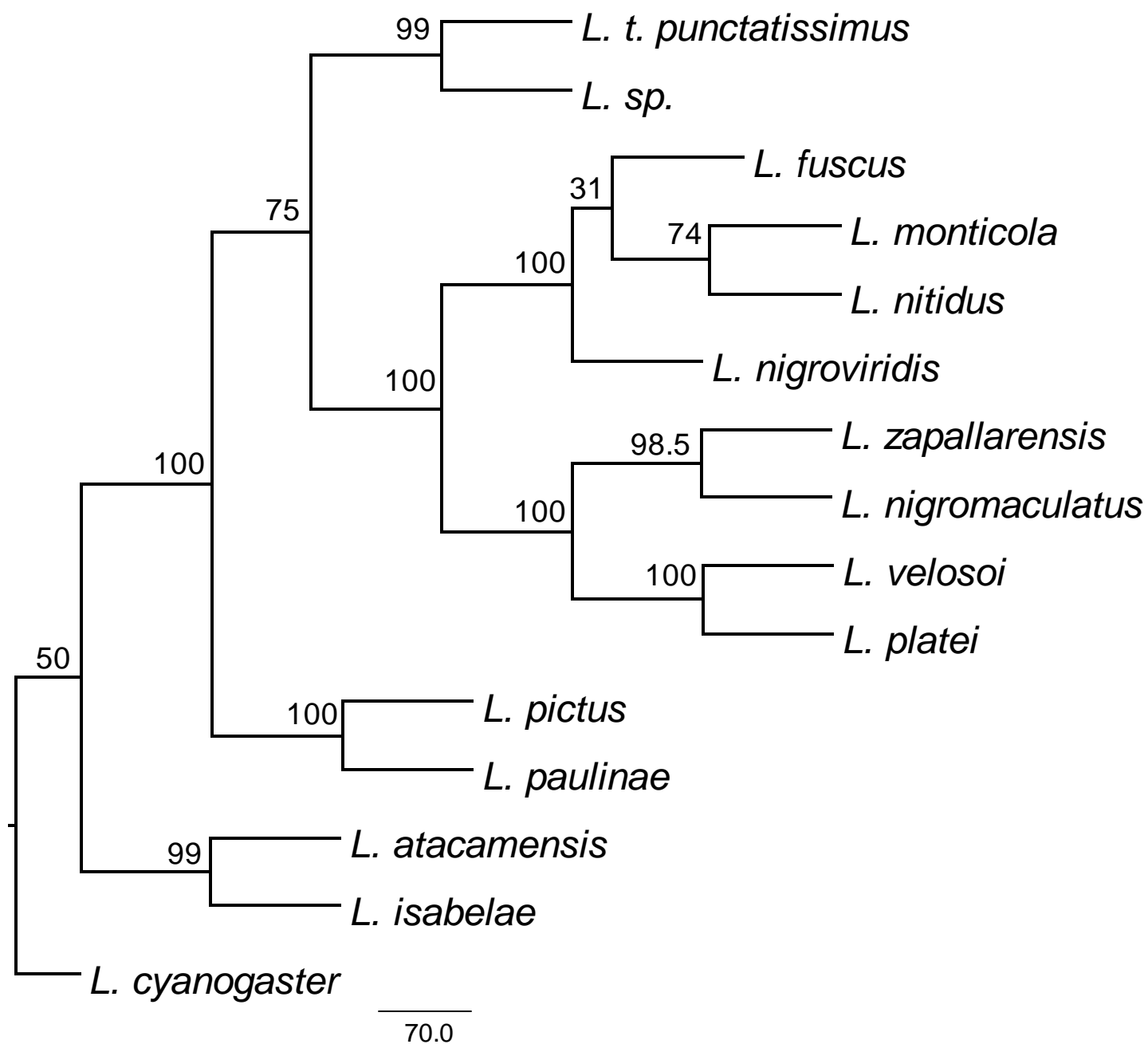

Supplement: Figure S2 — Values next to nodes indicate bootstrap support. [file peerj-05-3941-s008.pdf]

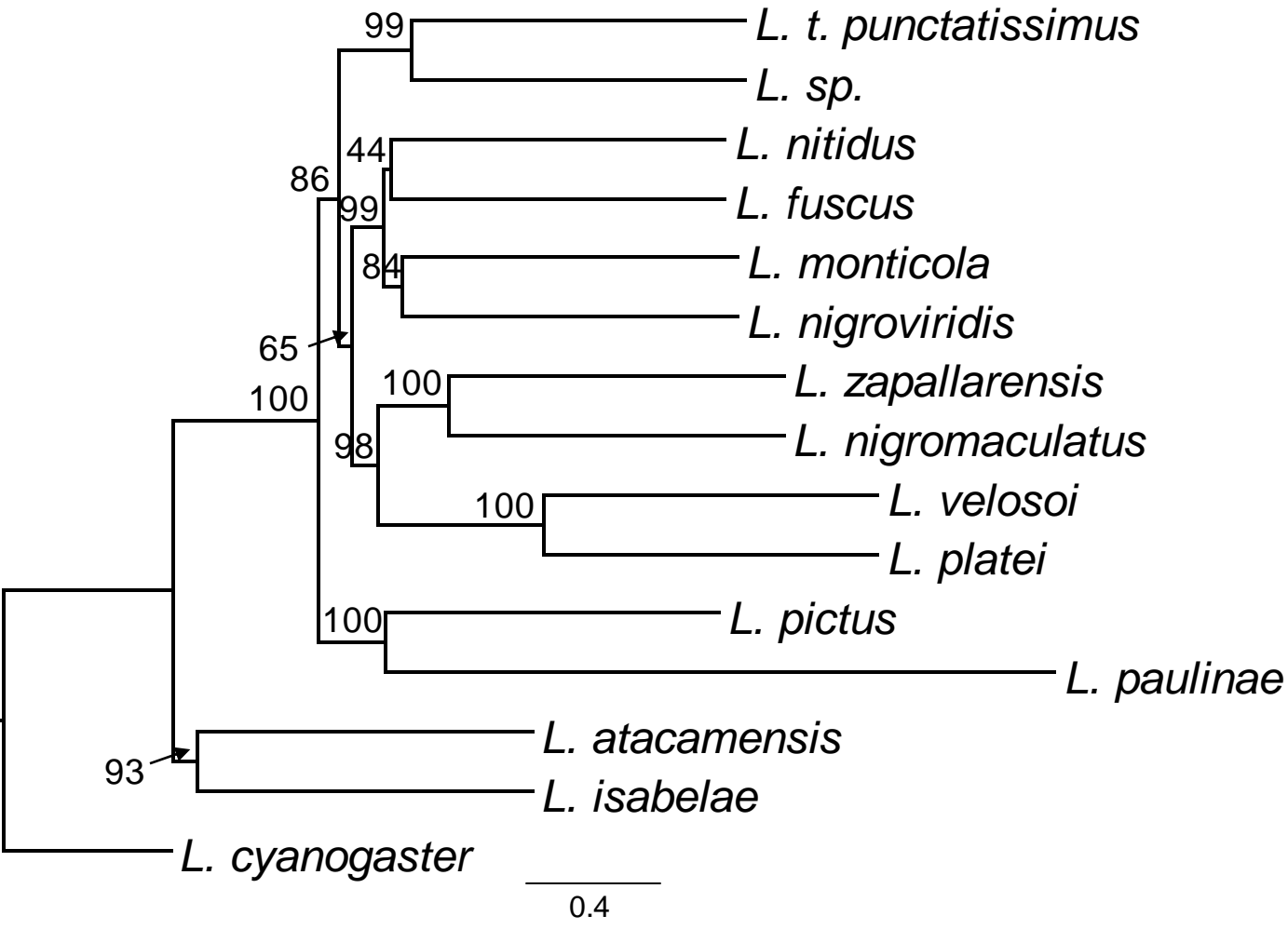

Supplement: Figure S3 — Values next to nodes indicate bootstrap support. [file peerj-05-3941-s009.pdf]
